# SOX4-BMI1 axis promotes non-small cell lung cancer progression and facilitates angiogenesis by suppressing ZNF24

**DOI:** 10.1038/s41419-024-07075-w

**Published:** 2024-09-30

**Authors:** Ting Wen, Xiao Zhang, Yun Gao, Hong Tian, Lufeng Fan, Ping Yang

**Affiliations:** 1 Department of Respiratory and Critical Care Medicine, The Second Affiliated Hospital of Shandong First Medical University, Taian, Shandong 271000 China; 2 Department of Internal Medicine, Shandong Provincial Taishan Hospital, Taian, Shandong 271000 China; 3 Department of General Surgery, Qilu Hospital (Qingdao), Cheeloo College of Medicine, Shandong University, Qingdao, Shandong 266035 China; 4 Department of Oncology, Qingdao Endocrine and Diabetes Hospital & Institute, Qingdao, Shandong 266000 China

**Keywords:** Non-small-cell lung cancer, Prognostic markers

## Abstract

The incidence of lung cancer has become the highest among all cancer types globally, also standing as a leading cause of cancer-related deaths. Lung cancer is broadly divided into small cell lung cancer (SCLC) and non-small cell lung cancer (NSCLC), with the latter accounting for 85% of total cases. SRY-box transcription factor 4 (SOX4), a crucial transcription factor, has been found to play a key role in the development of various cancers. However, the association between SOX4 and NSCLC is still unclear. This study investigated the clinical relevance of SOX4 and its potential mechanisms in the progression of NSCLC. Analysis of our NSCLC patient cohort revealed a significant increase in SOX4 levels in cancerous tissues, indicating its role as an independent prognostic indicator for NSCLC. In vitro experiments demonstrated that elevated SOX4 expression facilitated NSCLC cell migration, invasion, and EMT. Functionally, SOX4 drives NSCLC progression by enhancing the transcription and expression of B-cell-specific moloney leukemia virus insertion site 1 (BMI1). The oncogenic impact of SOX4-induced BMI1 expression on NSCLC advancement was validated through both in vivo and in vitro studies. In addition, our findings showed that BMI1 promoted the ubiquitination of histone H2A (H2Aub), leading to decreased zinc finger protein 24 (ZNF24) expression, which subsequently triggered vascular endothelial growth factor A (VEGF-A) secretion in NSCLC cells, thereby promoting NSCLC angiogenesis. Moreover, we evaluated the therapeutic potential of a BMI1 inhibitor in combination with Bevacizumab for NSCLC treatment using orthotopic models. The data presented in our study reveal a previously unrecognized role of the SOX4-BMI1 axis in promoting NSCLC progression and angiogenesis. This research significantly contributes to our knowledge of the interplay between SOX4 and BMI1 in NSCLC, potentially paving the way for the development of targeted therapies for this disease.

## Introduction

Lung cancer continues to be a significant factor in cancer-related deaths worldwide, ranking as the primary cause of cancer mortality in men and the second in women [1]. NSCLC accounts for the majority of cases, with lung squamous cell carcinoma (LUSC) and lung adenocarcinoma (LUAD) being the main subtypes [2]. Despite their distinct characteristics, similar treatment approaches are commonly used for both [3, 4]. Unfortunately, the prognosis for NSCLC patients remains grim due to late-stage detection and metastasis. Therefore, understanding the molecular mechanisms driving NSCLC progression is crucial for identifying diagnostic biomarkers and developing effective therapies [5].

The role of tumor-related transcription factors in cancer initiation and progression has recently become a focus of research. SOX4, a member of the SRY-related HMG-box gene family, is known for its highly conserved HMG domain that enables specific DNA binding [6, 7]. It has been identified as a common transcription factor in the progression and metastasis of various cancers, such as breast cancer, colorectal cancer, head and neck cancer, esophageal cancer, and cholangiocarcinoma [8–12]. Notably, SOX4 is also involved in the regulation of angiogenesis under pathological conditions [13, 14]. It has been reported that SOX4 promotes angiogenesis in glioblastoma or ovarian cancer by regulating VEGF-A [15, 16]. As an important factor in promoting tumor angiogenesis, researchers found that VEGF-A is highly expressed in lung cancer and is significantly related to the poor prognosis of lung cancer [17]. However, the specific role of SOX4 in NSCLC and its regulatory mechanism on VEGF-A are still not well understood.

BMI1, a member of the Polycomb group protein family, acts as a core component of Polycomb repressive complex 1 (PRC1) to facilitate gene silencing through histone H2A ubiquitination [18, 19]. Numerous studies have underscored its crucial role in cancer development by modulating gene expression programs associated with self-renewal, EMT, and metastasis [20–22]. There is a growing body of evidence supporting the significant anti-cancer effects of targeted therapy against BMI1 across different cancer types [23–25]. Specifically in the context of NSCLC, BMI1 inhibition has been shown to effectively reverse lung cancer progression [26]. Ongoing research is focused on unraveling the regulatory mechanisms of BMI1 expression and its downstream targets in NSCLC, representing an active area of exploration.

In our study, we systematically investigated the significance of SOX4 expression in a large cohort of NSCLC. Our findings revealed that SOX4 is significantly upregulated in NSCLC tissues, and patients with high SOX4 expression experience a poorer prognosis. In vitro experiments further supported the crucial role of SOX4 in the proliferation, migration, and invasion of NSCLC. Mechanistically, we clarified that BMI1 is directly regulated by the transcription of SOX4, and explored the correlation between SOX4 and BMI1 expression patterns and their prognostic implications. Through both in vivo and in vitro experiments, we elucidated the promoting effect of the SOX4-BMI1 axis on NSCLC progression. Interestingly, the study also uncovered the impact of the SOX4-BMI1 axis on angiogenesis in NSCLC. Our study demonstrated that BMI1 negatively regulates ZNF24 expression through a histone H2Aub-dependent mechanism, triggering VEGF-A secretion in NSCLC cells and promoting angiogenesis. Finally, the mouse orthotopic model demonstrated that co-administration of a BMI1 inhibitor with Bevacizumab enhances the anti-cancer efficacy of Bevacizumab in NSCLC, suggesting new avenues for clinical application.

## Materials and methods

### Human NSCLC samples

NSCLC tissue microarray (TMA) containing tumor and para-tumor tissues from 93 patients who underwent tumor resection surgery at the Second Affiliated Hospital of Shandong First Medical University from 2015 to 2020. Staging tumors according to the 8th edition of the AJCC/UICC TNM classification system and monitoring patients’ prognosis through follow-up. The Ethics Committee of the Second Affiliated Hospital of Shandong First Medical University has approved this study, and all patients involved in this study provided written informed consent.

### Xenograft model

Female BALB/c nude mice, aged 5–6 weeks, were obtained from GemPharmatech Co., Ltd. (Nanjing, China). Mice were randomly divided into four groups (*n* = 6). 5 × 10^6^ H1299 cells were injected subcutaneously into the right flanks of nude mice. After subcutaneous tumor formation, the body weight of the mice was measured every 4 days and the tumor diameter was measured every 2 days using an external caliper. Tumor volume (*V*) was calculated according to the formula as follows: *V* = (*L* × *W*^2^)/2, where *V* is the volume (mm^3^), *L* is the length (mm), and *W* is the width (mm). The tumors were excised, photographed, and weighed after 28 days. During the measurement process related to animal tumors, the measurer is blinded to the group assignments of the animals and only records the experimental results corresponding to the mouse ear tag numbers. All animal experiments were approved by the Medical Ethics Committee of Shandong First Medical University.

### Orthotopic model

Fluorescently labeled H1299 cells expressing Luciferase were utilized for the study. To establish an orthotopic model of NSCLC, 1 × 10^6^ H1299 cells were suspended in 20 μL of matrix gel and subsequently injected into the left lung of mice (*n* = 10). When deemed necessary, the BMI1 inhibitor PTC-209 (60 mg/kg/d, s.c.) or Bevacizumab (5 mg/kg/3d, i.v.) was administered starting from the second week after the injection of H1299 cells. The tumor was monitored by a live imaging system (IVIS Spectrum). Radiant efficiency was measured to quantify the tumor burden of mice. After four weeks, 6 mice were euthanized, and their lungs were dissected. The lung lobes were rinsed with cold PBS to remove surface blood, blotted dry with filter paper, and the wet lung weight was measured. Then, the right lung lobe was sectioned, and its largest cross-section was selected for H&E staining. The number of metastatic nodules was counted in this section. The remaining four mice were used to collect survival data. When the tumor burden became detrimental to their health, impaired mobility, or caused signs of imminent death, the mice were considered to have reached the endpoint. They were euthanized, and their survival times from the start of H1299 cell injection until death were recorded.

All animal experiments were approved by the Medical Ethics Committee of Shandong First Medical University.

### Statistical analysis

Statistical analyses were performed using SPSS 26.0 and GraphPad Prism 8.1 software. The sample size required for the experiment was carefully evaluated before the experiment began. Before conducting statistical analyses, the distribution of the obtained data, within-group variation, and variance between different comparison groups were analyzed and evaluated. Based on these assessments, an appropriate statistical analysis method was selected. The Chi-square test was used to evaluate the correlation between SOX4 or BMI1 expression and clinicopathological features. Survival curves were estimated using the Kaplan–Meier method and compared with the log-rank test, to analyze the prognostic significance of clinical and pathological features. The independent prognostic significance of clinicopathological characteristics was analyzed using multivariate analysis with the Cox proportional hazards regression model. One-way ANOVA, two-way ANOVA, or *t*-test was used to compare the statistical differences between groups. *p*-values < 0.05 were considered to be significant.

Other materials and methods are detailed in Supplementary Materials and Methods.

## Results

### SOX4 expression is significantly upregulated in NSCLC and is associated with a poor prognosis

**Fig. 1:**
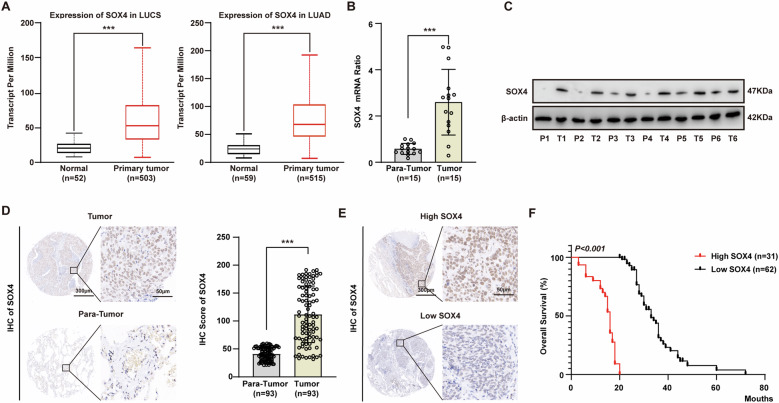
SOX4 expression is upregulated in NSCLC tissues and predicts a poor clinical outcome. **A** The mRNA levels of SOX4 in the LUSC (left) and LUAD (right) tissues, as well as their adjacent normal tissues, in the TCGA database. **B** The mRNA levels of SOX4 in 15 pairs of NSCLC tissues and para-tumor tissues were detected with qPCR. **C** The protein levels of SOX4 in 6 pairs of NSCLC tissues and para-tumor tissues were detected with western blot. **D** Left: Representative IHC images depicting the expression of SOX4 in NSCLC tissues and para-tumor tissues (left: scale bar, 300 μm; right: scale bar, 50 μm). Right: The IHC score of SOX4 was calculated between NSCLC tissues and para-tumor tissues. **E** Representative IHC images depicting the high or low expression of SOX4 in NSCLC tissues (left: scale bar, 300 μm; right: scale bar, 50 μm). **F** Overall survival curves of NSCLC patients were stratified by SOX4 expression. Patients with high SOX4 expression had significantly poorer overall survival (Kaplan–Meier method). *** represents *P* < 0.001. Data were analyzed with *t*-test (**A**), paired *t*-test (**B** and **D**), or log-rank test (**F**).

To elucidate the expression of SOX4 in NSCLC, we initially compared the mRNA levels of SOX4 in LUSC and LUAD tissues and their adjacent normal tissues in the TCGA database and found that the expression of SOX4 in tumor tissues was significantly higher than that in adjacent normal tissues (Fig. 1A). Subsequently, we used qPCR and western blot to determine the mRNA and protein levels of SOX4 in NSCLC tissues and adjacent normal tissues respectively, and also found that SOX4 was highly expressed in tumor tissues (Fig. 1B, C). Moreover, the expression of SOX4 was further detected with immunohistochemistry (IHC) in NSCLC tissue microarray (TMA). The IHC scoring further confirmed the significantly higher expression of SOX4 in NSCLC tissues compared to adjacent normal tissues (Fig. 1D).

To further assess the prognostic significance of SOX4 in NSCLC, the NSCLC patients were categorized into subsets with low/high SOX4 based on the cut-off value of IHC scores (Fig. 1E and Supplementary Table 1). The Kaplan–Meier method was used to analyze the correlation between SOX4 expression and corresponding clinical follow-up information. We found that high SOX4 expression was significantly associated with lower overall survival (OS) in NSCLC patients (*p* < 0.001) (Fig. 1F and Supplementary Table 3). These results show that SOX4 is an independent prognostic indicator of NSCLC.

### SOX4 significantly promotes the proliferation, migration, and invasion of NSCLC cells in vitro

**Fig. 2:**
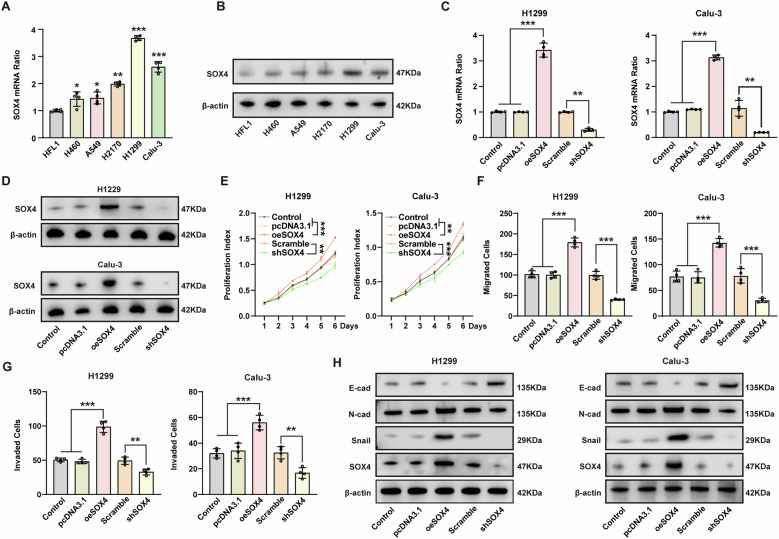
SOX4 promoted the proliferation, migration, and invasive capabilities of NSCLC cells. **A**, **B** The expression of SOX4 in the lung epithelial cell line HFL1 and different NSCLC cell lines was detected with qPCR (**A**) and western blot (**B**). **C**, **D** SOX4 was, respectively, overexpressed or knocked down in H1299 and Calu-3 cells, and the efficiency of knockdown was validated by qPCR (**C**) and western blot. **E** The proliferation levels of cells were determined by CCK-8 assay after overexpressing or silencing SOX4 in H1299 and Calu-3 cells. **F**, **G** The migration (**F**) and invasion (**G**) levels of cells were determined by transwell assay after overexpressing or silencing SOX4 in H1299 and Calu-3 cells. **H** The expression levels of EMT biomarkers were detected with western blot in H1299 and Calu-3 cells following SOX4 overexpression or knockdown. *, **, and *** represents *P* < 0.05, 0.01, and 0.001, respectively. Data were analyzed with paired *t*-test (**A**), one-way ANOVA (**C**, **F**, **G**), or two-way ANOVA (**E**). Data were from at least 3 independent experiments and shown as mean ± S.E.M.

The above results prompted us to explore the role of SOX4 in the progression of NSCLC. SOX4 expression was observed in various human NSCLC cell lines (H460, A549, H2170, H1299, Calu-3) as well as in the lung epithelial cell line HFL1. Results indicated lower mRNA and protein levels of SOX4 in HFL1 compared to NSCLC cell lines (Fig. 2A, B). Lentivirus-mediated overexpression or knockdown of SOX4 in H1299 and Calu-3 cells was confirmed through qPCR and western blot analysis (Fig. 2C, D). Subsequent evaluation of proliferation, migration, and invasion in these cells using CCK-8 and transwell assay revealed that SOX4 overexpression enhanced these capabilities, while knockdown had the opposite effect (Fig. 2E–G and Supplementary Fig. 1A, B). Previous research has suggested that SOX4 can induce EMT in various cancers, promoting aggressive invasion [8–12]. The study investigated the influence of SOX4 on EMT-related proteins like E-cadherin, N-cadherin, and Snail. Results showed that SOX4 overexpression reduced E-cadherin levels and increased N-cadherin and Snail expression, while SOX4 knockdown had the opposite effect on EMT markers (Fig. 2H), highlighting the role of SOX4 in promoting EMT. Overall, the findings demonstrate the crucial involvement of SOX4 in the proliferation and metastasis of NSCLC cells.

### SOX4 promotes the transcription and expression of BMI1 in NSCLC

**Fig. 3:**
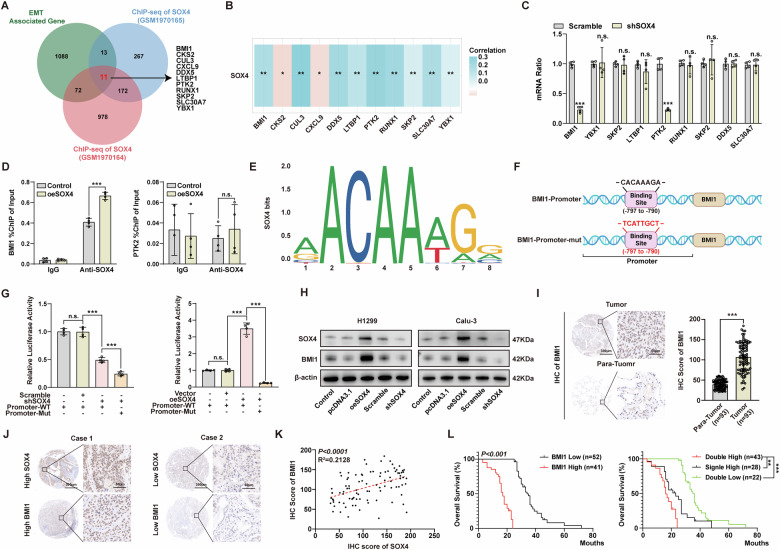
SOX4, as a transcription factor, regulates the expression of BMI1 in NSCLC. **A** Flowchart of screening downstream genes of SOX4 by the bioinformatics method. A total of 11 overlapping genes were screened out by the results in the EMT-associated gene sets and ChIP-seq datasets (GSM1970164 and GSM1970165). **B** Analysis of the correlation between the overlapping 11 genes and SOX4 in the TCGA database. **C** After knocking down SOX4 in H1299 cells, the expression changes of the 11 candidate genes were assessed using qPCR. **D** After overexpressing SOX4 in H1299 cells, the occupancy of SOX4 on the promoter regions of BMI1 or PTK2 was detected by ChIP-qPCR. **E** By Jaspar, the sequence with the highest predicted affinity for SOX4 was identified. **F** The sequence ‘CACAAAGA’ was located within the upstream promoter region of BMI1 at positions −797 to −790. Based on this sequence, a mutant unable to bind to SOX4 was constructed. **G** The transcriptional activity of BMI1 was detected in SOX4-silenced or SOX4-overexpressing H1299 cells, with or without the BMI1 promoter mutations, using the Dual-luciferase reporter assay. **H** After overexpressing or silencing SOX4 in H1299 and Calu-3 cells, BMI1 expression was detected using western blot. **I** Left: Representative IHC images depicting the expression of BMI1 in NSCLC tissues and para-tumor tissues (left: scale bar, 300 μm; right: scale bar, 50 μm). Right: The IHC score of BMI1 was calculated between NSCLC tissues and para-tumor tissues. **J** Representative IHC images depicting the expression of SOX4 and BMI1 in two cases of NSCLC tissues (left: scale bar, 300 μm; right: scale bar, 50 μm). **K** Correlation analysis of IHC scores for SOX4 and BMI1 in NSCLC tissues was conducted using the Pearson correlation test. **L** Left: Overall survival curves of NSCLC patients were stratified by BMI1 expression. Right: The overall survival curves were further stratified into subgroups based on the co-expression, single high expression, and double low expression of SOX4 and BMI1 (Kaplan–Meier method). n.s. represents not significant; *, **, and *** represents *P* < 0.05, 0.01, and 0.001, respectively. Data were analyzed with paired *t*-test (**C**, **D**, and **I**) or one-way ANOVA (**G**). Data were from at least 3 independent experiments and shown as mean ± S.E.M.

Multiple studies have demonstrated the transcriptional effects of SOX4 in various cancer progressions [8–12]. Building upon this foundation, we first explored the downstream targets through which SOX4 exerts its pro-carcinogenic effects in NSCLC. By overlapping three gene sets, including the ChIP-seq dataset (GSM1970164, GSM1970165) and EMT-associated genes, we identified 11 candidate genes regulated by SOX4 (Fig. 3A). Subsequent correlation analysis based on the expression levels of these 11 candidate genes and SOX4 in the TCGA database revealed that except for CKS2 and CXCL9, the other candidate genes exhibited a positive correlation with SOX4 (Fig. 3B). qPCR was employed to assess the changes in the expression of the remaining 9 candidate genes in H1299 cells after knocking down SOX4, and the results showed that only the mRNA levels of BMI1 and PTK2 were decreased (Fig. 3C).

To investigate the potential transcriptional regulatory effect of SOX4 on BMI1 and PTK2, we overexpressed SOX4 in H1299 cells and performed ChIP-qPCR experiments utilizing these SOX4-overexpressing H1299 cells to examine the interaction between the promoters of BMI1 and PTK2 and SOX4. Our results indicated a specific binding affinity of SOX4 towards the BMI1 promoter region, and this affinity was more pronounced in the SOX4 overexpression group (Fig. 3D). In addition, we queried the promoter region of the human BMI1 gene in the NCBI database and utilized JASPAR to predict the SOX4 binding affinity sequences within the BMI1 promoter region (Supplementary Table 4). Based on the query results, we constructed a nucleotide frequency matrix for the SOX4 binding affinity (Fig. 3E). For the potential SOX4 binding sequences identified, we conducted ChIP-qPCR and dual-luciferase reporter assays (Supplementary Fig. 2). Ultimately, we determined that SOX4 can specifically bind to the nucleotide sequence from -797 to -790 (CACAAAGA) in the BMI1 promoter region. Subsequently, plasmids containing the full BMI1 promoter region sequence and the BMI1 promoter region sequence with a mutated SOX4 binding site were designed for a dual-luciferase reporter assay (Fig. 3F). Knockdown of SOX4 resulted in a significant decrease in reporter gene activity, with a further reduction observed in the BMI1 promoter mutant. Conversely, overexpression of SOX4 led to a significant increase in reporter gene activity, whereas this enhancement was completely abolished when the mutated binding region plasmid was used (Fig. 3G).

To validate the impact of SOX4 on BMI1 expression, we modulated the expression of SOX4 in NSCLC cells. The results showed that overexpressing SOX4 led to an increase in BMI1 expression while knocking down SOX4 resulted in the opposite effect (Fig. 3H). Moreover, the expression of BMI1 was further detected with IHC in TMA of NSCLC. Similar to SOX4, the IHC scoring further confirmed the significantly higher expression of BMI1 in NSCLC tissues compared to adjacent normal tissues (Fig. 3I and Supplementary Table 2). To clarify the correlation between SOX4 and BMI1 expression, we performed IHC on the NSCLC tissue of the same patient and found that patients with high SOX4 expression also had high expression of BMI1, while patients with low SOX4 expression also had low expression of BMI1 (Fig. 3J). Meanwhile, there was a significant positive correlation between the IHC scores of SOX4 and BMI1 (Fig. 3K). The above results indicate that SOX4 regulates the transcription and expression of BMI1 in NSCLC.

### Co-expression of SOX4 and BMI1 correlated with the most unfavorable prognosis of NSCLC

We further investigated the prognostic significance of BMI1 in NSCLC and observed a strong correlation between high BMI1 expression and lower overall survival (OS) in NSCLC patients, as evidenced by Kaplan–Meier analysis (*p* < 0.001), indicating that BMI1 is also a prognostic indicator for NSCLC (Fig. 3L left panel). In addition, we divided NSCLC patients into three subgroups based on their expression levels: double-high (co-expression of SOX4 and BMI1), single-high (high expression of either SOX4 or BMI1), and double-low (low expression of both SOX4 and BMI1). Our findings revealed that patients in the double-high subgroup had significantly worse prognosis compared to those in the single-high and double-low subgroups (*p* < 0.001) (Fig. 3L right panel and Supplementary Table 3). These results highlight the potential of using a combination of SOX4 and BMI1 to improve the accuracy of prognostic predictions in NSCLC patients, suggesting a potential joint role of SOX4 and BMI1 in the progression and prognosis of NSCLC.

### BMI1 was an important effector in SOX4-induced proliferation and metastasis of NSCLC

**Fig. 4:**
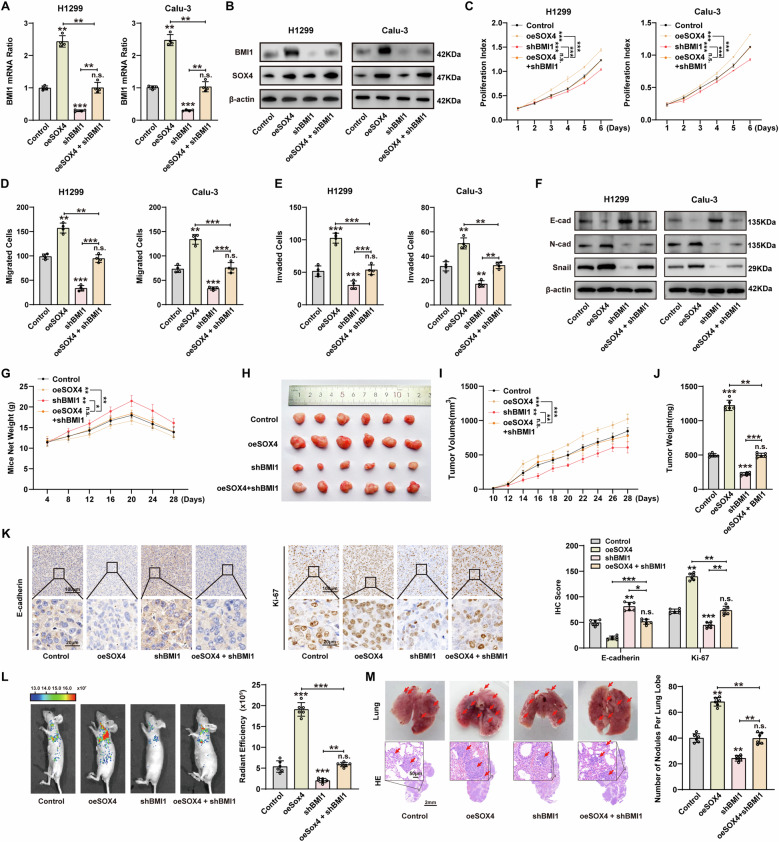
BMI1 was required in SOX4-induced proliferation and metastasis of NSCLC. **A**, **B** After overexpressing SOX4 or simultaneously knocking down BMI1 in H1299 and Calu-3 cells, BMI1 expression was detected using qPCR (**A**) and western blot (**B**). **C** The proliferation levels of cells were determined by CCK-8 assay after overexpressing SOX4 or simultaneously knocking down BMI1 in H1299 and Calu-3 cells. **D**, **E** The migration (**D**) and invasion (**E**) levels of cells were determined by transwell assay after overexpressing SOX4 or simultaneously knocking down BMI1 in H1299 and Calu-3 cells. **F** The expression levels of EMT biomarkers were detected with western blot in H1299 and Calu-3 cells after overexpression of SOX4 or simultaneous knockdown of BMI1. **G** Subcutaneous xenograft models were established in nude mice using stable SOX4 overexpression, BMI1 silencing, and simultaneous stable SOX4 overexpression with BMI1 silencing in H1299 cells. The net weight of mice was measured every 4 days. **H** The diameter of xenograft tumors was measured 28 days later. **I** The volume of xenograft tumors was recorded from day 10 to day 28. **J** The weights of xenograft tumors were measured after 28 days. **K** Representative IHC images and IHC score of E-cadherin and Ki-67 expression in xenograft tumors. upper: scale bars, 100 μm; lower: scale bars, 20 μm. **L** Orthotopic model in nude mice was established with stable SOX4 overexpression, BMI1 silencing, and simultaneous stable SOX4 overexpression with BMI1 silencing in H1299 cells. The tumor metastases were monitored by a live imaging system (left). Radiant efficiency of in vivo fluorescence was measured to quantify the tumor burden of mice (right). **M** Representative images of tumors and H&E staining of lung lesions (upper: scale bars, 50 μm; lower: scale bars, 2 mm) from the orthotopic model (left). The number of metastatic nodules in the lung was counted (right). n.s. represents not significant; *, **, and *** represents *P* < 0.05, 0.01, and 0.001 respectively. Data were analyzed with paired *t*-test (**A**, **D**, **E**, **J**, **K**, **L**, and **M**) or two-way ANOVA (**C**, **G**, and **I**). Data were from at least 3 independent experiments and shown as mean ± S.E.M.

The above results confirmed that SOX4 can regulate the expression and transcription of BMI1 in NSCLC, so we further studied the role of BMI1 in SOX4-induced NSCLC progression. We observed that BMI1 expression is upregulated in NSCLC cells overexpressing SOX4, but rescued by BMI1 knockdown (Fig. 4A, B and Supplementary Fig. 3A, B). Both CCK8 and transwell assay showed that knocking down BMI1 attenuated SOX4-induced proliferation, invasion, and migration of NSCLC cells (Fig. 4C–E and Supplementary Fig. 3C, D). We further investigated the impact of SOX4-mediated BMI1 expression on EMT biomarkers. The results showed that knocking down BMI1 inhibited the SOX4-induced EMT of NSCLC cells (Fig. 4F).

Then, we studied the role of SOX4-induced BMI1 expression in tumor proliferation and metastasis by establishing subcutaneous and orthotopic models in mice. Regarding tumor proliferation, stable SOX4 overexpression, BMI1 silencing, SOX4 overexpression while silencing BMI1, or control H1299 cells were subcutaneously injected into BALB/c nude mice. The net weight of mice in the overexpression SOX4 group was significantly lower than that of the control group while knocking down BMI1 abolishes this trend (Fig. 4G). The diameter, volume, and weight of overexpressed SOX4 xenografts were notably higher than those of the control group while knocking out BMI1 eliminated this trend (Fig. 4H–J). IHC detects the expression levels of E-cadherin and Ki-67 in xenografts to evaluate their EMT and proliferation levels. The IHC scores indicate that E-cadherin exhibits low expression in SOX4-overexpressing xenografts, while Ki-67 shows relatively high expression levels while knocking out BMI1 similarly abolished this trend (Fig. 4K). In terms of tumor metastasis, fluorescently labeled H1299 cells were injected into the left lung of BALB/c nude mice. The radiant efficiency of in vivo fluorescence was measured to quantify the tumor burden of mice. The in vivo fluorescence radiation efficiency of mice in the SOX4 overexpression group was significantly increased, and knocking down BMI1 weakened this trend (Fig. 4L). Subsequently, the mice were euthanized, and tumor burden in the lungs was evaluated by measuring the wet lung weight and conducting H&E staining on the largest cross-section of the right lung lobe. The results showed that SOX4 overexpression significantly increased both the wet lung weight and the number of metastatic lesions, while simultaneous BMI1 knockdown rescued this effect (Fig. 4M and Supplementary Fig. 4A, B). For the remaining mice, we continued to monitor them and recorded their survival times. The results showed that the survival time was significantly shortened in the SOX4 overexpression group, while simultaneous BMI1 knockdown delayed the time of death (Supplementary Fig. 4C). The above results suggested that BMI1 was a key effector in SOX4-involved NSCLC progression.

### BMI1 inhibited the expression of ZNF24 by promoting histone H2Aub in NSCLC

**Fig. 5:**
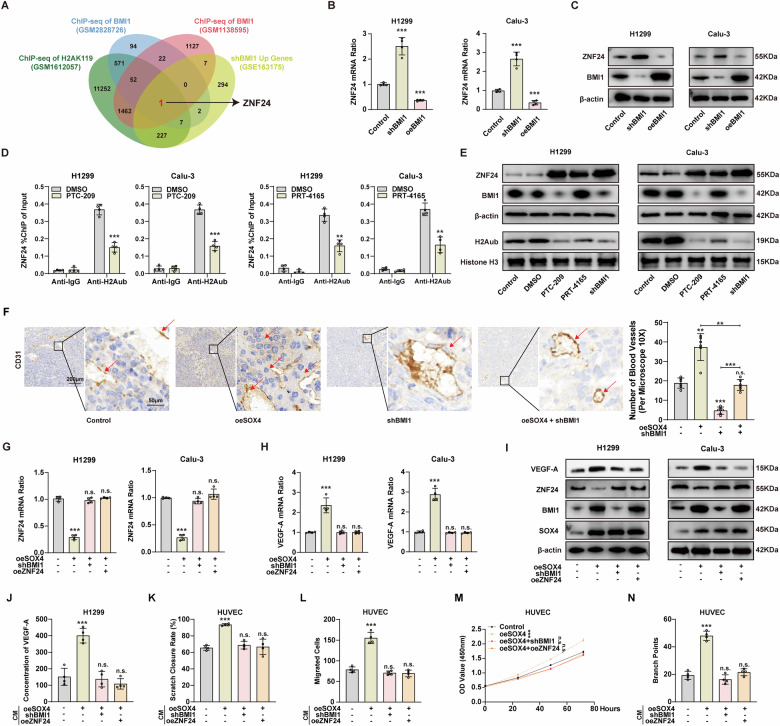
SOX4-BMI1 axis enhances angiogenesis in NSCLC by promoting the secretion of VEGF-A through inhibiting ZNF24 expression. **A** Flowchart of screening downstream genes of BMI1 by the bioinformatics method. The ZNF24 gene was identified through analysis of the ChIP-seq datasets (GSM2828726, GSM1612057, and GSM1138595) and RNA-seq results (GSE163175). **B**, **C** After knocking down or overexpressing BMI1 in H1299 and Calu-3 cells, ZNF24 expression was assessed using qPCR (**B**) and western blot (**C**). **D** The ChIP-qPCR assay showed that BMI1 inhibitor PTC-209 (10 μM) or H2Aub inhibitor PRT-4165 (10 μM) reduced H2Aub levels on the promoter of ZNF24 in H1299 and Calu-3 cells. **E** H1299 and Calu-3 cells were treated with PTC-209 (10 μM), PRT-4165 (10 μM), or knocking down BMI1, the ZNF24 and H2Aub expression were detected by western blot. **F** Left: Representative IHC images of CD31 expression in the orthotopic model (left: scale bars, 200 μm; right: scale bars, 50 μm). Right: Blood vessels were counted to estimate angiogenesis. **G**, **H** After overexpressing SOX4 and either knocking down BMI1 or overexpressing ZNF24 in H1299 and Calu-3 cells, ZNF24 (**G**) or VEGF-A (**H**) mRNA expression was detected using qPCR. **I** After overexpressing SOX4 and either knocking down BMI1 or overexpressing ZNF24 in H1299 and Calu-3 cells, VEGF-A, ZNF24, BMI1, and SOX4 expression were detected using western blot. **J** After overexpressing SOX4 and either knocking down BMI1 or overexpressing ZNF24 in H1299 cells, the concentration of VEGF-A in the CM was determined using ELISA. **K**–**M** HUVEC cells were incubated with CM from H1299 cells. The healing (**K**), migration (**L**), and proliferation (**M**) capabilities of HUVEC cells were evaluated. **N** HUVEC cells were incubated with CM from H1299 cells. Vascular tube formation was evaluated based on branch points. n.s. represents not significant; *, **, and *** represents *P* < 0.05, 0.01, and 0.001, respectively. Data were analyzed with paired *t*-test (**B**, **D**, **F**, **G**, **H**, **J**–**L**, and **N**) or two-way ANOVA (**M**). Data were from at least 3 independent experiments and shown as mean ± S.E.M.

It is known that BMI1 mainly promotes the ubiquitination of histone H2A, thus exerting its transcriptional repressive effect [18, 19]. Inspired by this conclusion, we first explored the downstream targets through which BMI1 exerts its pro-carcinogenic effects in NSCLC. By overlapping four gene sets, including the ChIP-seq dataset (GSM2828726, GSM1612057, and GSM1138595) and RNA sequencing results (GSE163175), we finally identified ZNF24, the downstream target of BMI1 (Fig. 5A). Subsequently, qPCR and western blot were used to verify the expression changes of ZNF24 in NSCLC cells after knocking down or overexpressing BMI1. The results showed that the mRNA and protein levels of ZNF24 were negatively correlated with BMI1, suggesting that ZNF24 may be negatively regulated by BMI1 (Fig. 5B, C).

We further investigated the potential inhibitory effect of BMI1 on the transcription of ZNF24 through H2Aub. NSCLC cells were treated with BMI1 inhibitor PTC-209 or H2Aub inhibitor PRT-4165, followed by ChIP-PCR analysis to examine the interaction between the ZNF24 promoter and H2Aub. The results indicated a significant reduction in H2Aub levels on the ZNF24 promoter upon treatment with PTC-209 or PRT-4165 (Fig. 5D). Furthermore, individual treatment of NSCLC cells with PTC-209 and PRT4165 led to a decrease in H2Aub levels and an increase in ZNF24 expression (Fig. 5E). These findings suggest that BMI1 suppresses the transcription and expression of ZNF24 in NSCLC cells by enhancing repressive H2Aub marks on the ZNF24 promoter.

### SOX4-BMI1 axis promotes angiogenesis in NSCLC by inhibiting the expression of ZNF24

The role of SOX4 in promoting tumor angiogenesis is well-established, but its specific impact on NSCLC angiogenesis remains unclear. Previous studies have shown that ZNF24 can inhibit tumor angiogenesis by reducing the expression of VEGF-A [27, 28]. Therefore, our study aims to investigate whether the SOX4-BMI1 axis promotes angiogenesis in NSCLC by inhibiting the expression of ZNF24. IHC analysis of mouse tumor tissue in Fig. 4 revealed that overexpression of SOX4 significantly increased the number of blood vessels in the tumor, while knockout of BMI1 reversed this effect (Fig. 5F). Furthermore, we employed Masson’s staining to visualize red blood cells within the lumens of tumor blood vessels, serving as an indirect method to evaluate angiogenesis (Supplementary Fig. 5). Following overexpression of SOX4 in NSCLC cells, there was an observed increase in the mRNA or protein level of ZNF24. However, this trend was reversed when BMI1 was knocked down or when ZNF24 was overexpressed. Interestingly, the changing trend of VEGF-A mRNA or protein level was found to be opposite to that observed with ZNF24 (Fig. 5G–I and Supplementary Fig. 6A, B). Interestingly, ELISA results showed that when NSCLC cells overexpressed SOX4, the concentration of VEGF-A in the CM (conditioned medium) increased, and this trend was reversed when BMI1 was knocked out or ZNF24 was overexpressed (Fig. 5J).

To investigate the role of the SOX4-BMI1 axis in NSCLC vasculature, we analyzed the impact of the mentioned CM on the healing, migration, and proliferation capacities of HUVEC. In addition, we assessed angiogenesis using a vascular tube formation assay. The overexpression of SOX4 in the CM led to a significant enhancement in HUVEC healing, migration, proliferation, and angiogenesis. As predicted, this effect was reversed upon knockout of BMI1 or overexpression of ZNF24 (Fig. 5K–N and Supplementary Fig. 7A–D). The above results indicate that the SOX4-BMI1 axis promotes the expression and secretion of VEGF-A in NSCLC cells by inhibiting the expression of ZNF24, ultimately increasing angiogenesis.

### The blockade of BMI1 enhances the therapeutic efficacy of Bevacizumab in the NSCLC orthotopic model

**Fig. 6:**
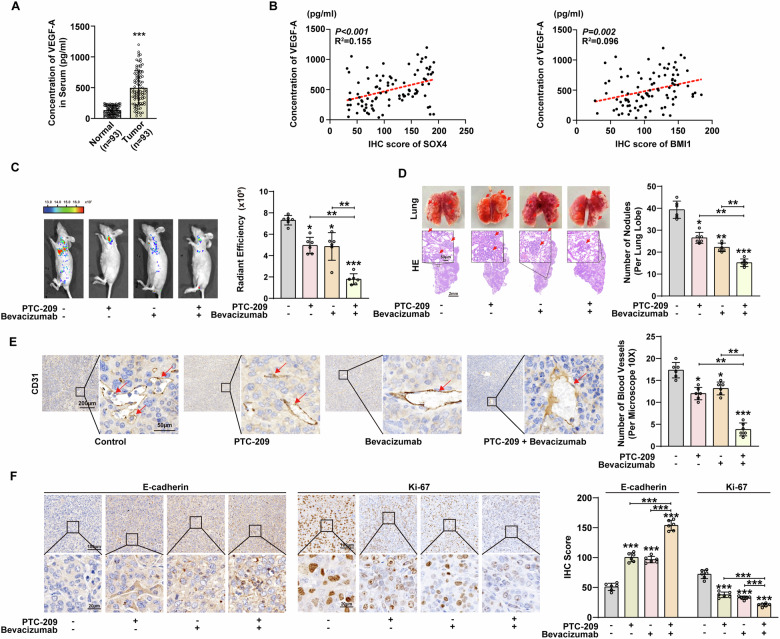
The blockade of BMI1 enhances the anti-tumor effects of Bevacizumab in NSCLC patients. **A** The concentration of VEGF-A in the serum of NSCLC patients and healthy individuals was detected with ELISA. **B** Correlation between the IHC scores of SOX4 or BMI1 in NSCLC tissues and the concentration of VEGF-A in serum was conducted using the Pearson correlation test. **C** Orthotopic model in nude mice was established with H1299 cells. After two weeks, PTC-209 (60 mg/kg/d, s.c.) or Bevacizumab (5 mg/kg/3d, i.v.) was used. The tumor metastases were monitored by a live imaging system (left). Radiant efficiency of in vivo fluorescence was measured to quantify the tumor burden of mice (right). **D** Representative images of tumors and H&E staining of lung lesions (upper: scale bars, 50 μm; lower: scale bars, 2 mm) from the orthotopic model (left). The number of metastatic nodules in the lung was counted (right). **E** Left: Representative IHC images of CD31 expression in the orthotopic model (left: scale bars, 200 μm; right: scale bars, 50 μm). Right: Blood vessels were counted to estimate angiogenesis. **F** Representative IHC images and IHC score of E-cadherin and Ki-67 expression in orthotopic model. upper: scale bars, 100 μm; lower: scale bars, 20 μm *, **, and *** represents *P* < 0.05, 0.01, and 0.001 respectively. Data were analyzed with paired *t*-test (**A**, **C**, **D**, **E**, and **F**). Data were from at least 3 independent experiments and shown as mean ± S.E.M.

Next, we evaluated the concentration of VEGF-A in the serum of NSCLC patients and healthy donors and found that the concentration of VEGF-A in the serum of the former was significantly elevated (Fig. 6A). Meanwhile, the concentration of VEGF-A in the serum of NSCLC patients was positively correlated with the expression levels of SOX4 or BMI1 in tumors (Fig. 6B).

In a previous phase III clinical trial, it was found that adding Bevacizumab to standard first-line chemotherapy significantly improved progression-free survival (PFS) in patients with advanced NSCLC [29]. However, emerging evidence suggests that tumor cells can evade anti-angiogenic treatments through the production of VEGF-A, a mechanism that may limit the therapeutic efficacy of Bevacizumab in NSCLC [30]. BMI1 inhibitors have found application in the clinical treatment of various tumors [31, 32]. Therefore, we used fluorescently labeled H1299 cells to establish an orthotopic model in BALB/c mice to study whether BMI1 inhibitors can further enhance the anti-tumor effect of Bevacizumab. Compared with the control group, the fluorescence radiation efficiency in mice treated with PTC-209 or Bevacizumab alone was weakened, while combined treatment resulted in a significant reduction in fluorescence radiation efficiency (Fig. 6C). Consistently, H&E staining and wet lung weight measurements demonstrated that the combination treatment significantly reduced the number of metastatic lesions (Fig. 6D and Supplementary Fig. 8A, B). Furthermore, by documenting the survival times of the remaining mice, we observed that the combination treatment significantly prolonged their survival (Supplementary Fig. 8C). The CD31 IHC staining and Masson’s staining results also showed that the combined treatment of PTC-209 and Bevacizumab can effectively inhibit tumor angiogenesis, and its efficacy is better than single drug treatment (Fig. 6E and Supplementary Fig. 9). Similarly, IHC results show that compared with single drug treatment, combined treatment with PTC-209 and Bevacizumab can effectively promote the expression of E-cadherin and inhibit the expression of Ki-67, indicating that combined treatment can effectively reduce the EMT and proliferation levels of NSCLC (Fig. 6F). The lack of efficacy of Bevacizumab alone in the treatment of NSCLC may be because BMI1 promotes the secretion of VEGF-A by NSCLC cells. These results confirm the role of BMI1 inhibitor in enhancing the efficacy of targeted drugs in NSCLC and clarify its clinical therapeutic potential.

## Discussion

NSCLC is the most common type of lung cancer, and its incidence is increasing globally. Although the treatment options for NSCLC continue to improve, its five-year OS is still low, which poses a great challenge to clinical treatment [1]. For patients with early-stage NSCLC, surgical resection is one of the preferred treatments. Unfortunately, many cases are diagnosed at an advanced stage, thus limiting the therapeutic potential of surgery. For patients with advanced disease or those who have relapsed after surgery, chemotherapy becomes a key component of the treatment plan. However, the overall prognosis of NSCLC remains dismal because NSCLC patients vary in their response to chemotherapy and are prone to develop drug resistance [33]. In recent years, with in-depth research on tumor biology and molecular mechanisms, new treatment strategies such as immunotherapy and targeted therapy have continued to emerge, bringing hope to the majority of NSCLC patients [34]. It is well known that the development of new drug targets or treatments is usually based on the discovery of new biomarkers, which relies heavily on large-sample cohort studies. To address this challenge, we established a cohort of 93 NSCLC patients for whom tissue samples and follow-up data were available.

SOX4, a crucial transcription factor, exhibits high expression in various cancers and plays a significant role in controlling tumor cell proliferation, invasion, and metastasis [8–12]. Knocking down SOX4 has been shown to attenuate AKT and β-catenin activities, leading to a decrease in the invasive characteristics of uterine carcinosarcoma [6, 35]. In hepatocellular carcinoma, SOX4 suppresses p53-mediated apoptosis through transcriptional regulation of p53 [36]. In the context of NSCLC, SOX4 also exerts a cancer-promoting effect that cannot be ignored. Previous studies have confirmed that SOX4 is highly expressed in NSCLC samples and is associated with poor prognosis in patients [37]; Sun J et al. reported that the SOX4/β-catenin axis promotes the self-renewal of lung tumor cells, thereby driving the progression of lung cancer [38]; Chang J et al. confirmed that miR-363-3p inhibits NSCLC invasion and EMT by regulating SOX4 [39]. In this study, we confirmed that SOX4 is an adverse prognostic biomarker for NSCLC and a key factor promoting the progression of NSCLC.

A consensus has been reached on the prognostic significance and cancer-promoting effect of SOX4 in NSCLC, but its downstream targets and specific mechanisms need to be further clarified. Through bioinformatics analysis and other methods, we identified BMI1 as the downstream target gene of SOX4. Existing studies have also conducted preliminary exploration into the role of BMI1 in NSCLC progression and chemotherapy drug resistance [40, 41]. In this study, we observed a significant negative correlation between the expression of SOX4 and BMI1 in NSCLC tissues, and patients with high expression of both had worse prognoses. At the same time, our results show for the first time that BMI1 is an important effector of SOX4-induced NSCLC progression. It can be seen that the detection of SOX4 and BMI1 can stratify high-risk NSCLC patients and is expected to guide individualized treatment. Therefore, an in-depth study of the mechanism of action of the SOX4-BMI1 axis and exploration of therapeutic strategies targeting it is expected to open up new avenues for the treatment of NSCLC.

Tumor angiogenesis plays a crucial role in tumor growth and metastasis, being regulated by various factors. Among these, the VEGF family stands out as the most significant. VEGF-A, a key member of this family, facilitates tumor angiogenesis and progression by stimulating endothelial cell proliferation, migration, and lumen formation, while also enhancing vascular permeability [42]. It is well known that BMI1 mediates gene silencing mainly by promoting the ubiquitination of histone H2A and plays an important role in the occurrence and progression of various tumors [43, 44]. Here, we found that BMI1 inhibits ZNF24 expression by promoting H2Aub. Previous studies have confirmed that SOX4 can promote the expression of VEGF-A, but its specific mechanism has not been fully elucidated [15, 16]. Our discovery fills this mechanistic gap, and ZNF24 becomes a bridge connecting the SOX4-BMI1 axis and VEGF-A. ZNF24 has been shown to inhibit the transcription of VEGF-A [27, 28]. We verified this regulatory relationship in NSCLC and further confirmed that the SOX4-BMI1 axis promotes the secretion of VEGF-A by NSCLC cells by inhibiting the expression of ZNF24, thereby promoting the angiogenesis of NSCLC. This finding enhances our understanding of the SOX4-BMI1 axis in promoting NSCLC progression, making it a highly potential therapeutic target.

Currently, treating and preventing NSCLC metastasis remains a significant challenge. Bevacizumab was first approved by the US FDA in 2004 for the treatment of colorectal cancer. Subsequently, in 2008, the FDA approved its use in the treatment of NSCLC, but cases of drug resistance continue to be reported [45, 46]. How to effectively solve the problem of Bevacizumab resistance during the treatment of NSCLC is in the exploratory stage [47, 48]. Notably, the widespread involvement of BMI1 in cancer provides a possibility for targeted therapy against BMI1 in human malignancies. BMI1 inhibitors, such as PTC-209 and PTC-596, are currently being evaluated in clinical trials for the treatment of solid tumors [31, 49]. This study confirmed through the mouse orthotopic NSCLC model that compared with Bevacizumab alone, the combination of BMI1 inhibitor PTC-209 and Bevacizumab showed better tumor suppression effect. Our study provides new insights into solving the problem of Bevacizumab resistance in NSCLC treatment and also provides theoretical support for exploring new drug targets and combination treatments for NSCLC.

**Fig. 7:**
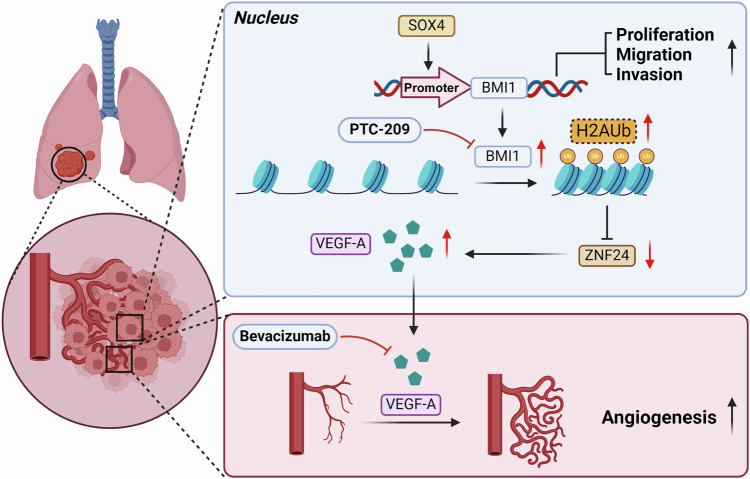
Schematic depiction of the mechanisms underlying SOX4-BMI1 axis facilitating NSCLC progression and angiogenesis. SOX4, as a transcription factor, promotes the transcription and expression of BMI1 in NSCLC, significantly enhancing tumor growth and metastasis. In addition, BMI1 promotes H2Aub, suppressing the expression of ZNF24 in NSCLC. The latter, by increasing the secretion of VEGF-A in NSCLC cells, leads to an increase in vascular generation in the TME, further promoting the progression of NSCLC.

In conclusion, we systematically investigated the regulatory role of the SOX4-BMI1 axis in NSCLC, unveiling a specific mechanism: SOX4 is highly expressed in NSCLC and promotes the transcription and expression of BMI1. This interaction between SOX4 and BMI1 contributes to tumor growth and metastasis. In addition, BMI1 facilitates the ubiquitination of histone H2A, resulting in the suppression of ZNF24 expression. This suppression, in turn, enhances tumor angiogenesis by upregulating the secretion of VEGF-A in NSCLC cells, further driving NSCLC progression (Fig. 7). Our research highlights how the SOX4-BMI1 axis not only mediates NSCLC advancement but also plays a role in conferring resistance to Bevacizumab. Therefore, the SOX4-BMI1 axis can serve as a promising drug target in NSCLC.

## Supplementary information

Supplementary Materials, Figures, and Tables

Western Blot Original Image

## Data Availability

The datasets used and/or analyzed during the current study are available from the corresponding author upon reasonable request.
