# Supplementary material for: SOX4-BMI1 axis promotes non-small cell lung cancer progression and facilitates angiogenesis by suppressing ZNF24
Source: Cell Death Dis. 2024 Sep 30;15(9):698. doi: 10.1038/s41419-024-07075-w (PMC11442842; doi:10.1038/s41419-024-07075-w)
Supplement: Supplementary file 1 — Supplementary Materials, Figures, and Tables [file 41419_2024_7075_MOESM1_ESM.docx]

Supplementary Materials, Figures, and Tables of

**SOX4-BMI1 Axis Promotes Non-Small Cell Lung Cancer Progression and Facilitates Angiogenesis**

**by Suppressing ZNF24**

Ting Wen et al.

**Supplementary Materials and Methods**

**Cell lines**

The human lung cancer cell lines H460, A549, H1299, and Calu-3, the lung epithelial cell line HFL1, and the human umbilical vein endothelial cell line HUVEC all originate from the Cell Bank of the Chinese Academy of Sciences (Shanghai, China). The human lung cancer cell line H2170 was acquired from the American Type Culture Collection (Manassas, VA, USA). HUVEC cells were cultured in ECM medium (Gibco, New York, USA), while other cell lines were maintained in DMEM medium (Gibco) supplemented with 10% FBS (Gibco) and penicillin/streptomycin (Thermo Fisher, Waltham, MA, USA) in a humidified atmosphere containing 5% CO_2_. Each cell line was authenticated using STR analysis, with the Chinese Academy of Sciences or ATCC databases employed as references.

**Antibodies and reagents**

The main antibodies and reagents are as follows:

| **Antibodies** | **Source** | **Identifier** |
| --- | --- | --- |
| SOX4 | Sigma-Aldrich | Cat. No. AV38234 |
| BMI1 | Cell Signaling Technology | Cat. No. D20B7 |
| E-cadherin | Cell Signaling Technology | Cat. No. 24E10 |
| N-cadherin | Cell Signaling Technology | Cat. No. D4R1H |
| Snail | Cell Signaling Technology | Cat. No. C15D3 |
| Ki-67 | Cell Signaling Technology | Cat. No.8D5 |
| Ubiquityl-Histone H2A  (Lys119) | Cell Signaling Technology | Cat. No. D27C4 |
| Histone H3 | Cell Signaling Technology | Cat. No. D1H2 |
| SOX4 | Abcam | Cat. No. Ab86809 |
| CD31 | Abcam | Cat. No. Ab28364 |
| VEGF-A | Abcam | Cat. No. Ab46154 |
| ZNF24 | Abcam | Cat. No. Ab176589 |
| β-actin | Abcam | Cat. No. Ab8226 |
| **Chemicals** | **Source** | **Identifier** |
| PRT-4165 | Selleck Chemicals | Cat. No. 31083-55-3 |
| PTC-209 | Selleck Chemicals | Cat. No. 315704-66-6 |
| Bevacizumab | Selleck Chemicals | Cat. No. A2006 |

Other reagents without special instruction are purchased from Sigma-Aldrich (Burlington, MA, USA).

**IHC and scoring**

For immunohistochemistry (IHC), the slides were deparaffinized and pretreated in either sodium citrate buffer (pH 6.0) or EDTA buffer (pH 9.0). Next, they were incubated overnight at 4°C with primary antibodies of SOX4 (1:200), BMI1 (1:200), CD31 (1:50), E-cadherin (1:400), or Ki-67 (1:800). Corresponding secondary immunoglobulin antibodies (Zsbio, Beijing, China) were applied for 30 minutes at 37°C. The slides were then incubated by binding to horseradish peroxidase streptavidin and developed with a 3,3-diaminobenzidine solution (Zsbio).

The Quant Center software quantified the IHC results of the tumor area. Staining intensity was categorized as weak, moderate, or strong, with the area of each staining calculated. The IHC score was determined as follows: IHC score = (percentage of cells with weak intensity×1) + (percentage of cells with moderate intensity×2) + (percentage of cells with strong intensity×3). The cohort was stratified into different groups based on the cut-offs of IHC scores, which were validated as the points with the highest combined specificity and sensitivity in the receiver operating characteristic (ROC) curves.

**Quantitative real-time PCR**

The RNAs from tissues and cells were extracted following the TRIzol (Thermo Fisher) manual's guidelines. A reverse transcriptase kit (TOYOBO, Osaka, Japan), SYBR Green Master Mix (Roche, Switzerland), and a Light Cycler Roche 480 PCR instrument were used to synthesize cDNA and conduct real-time PCR.

The relative expression levels of target genes were determined using the 2^-ΔΔCt^ method, with β-actin as an internal control. The primers employed for qPCR were listed in the following table:

| **Genes** | **Forward primer (5'- 3')** | **Reverse primer (5'- 3')** |
| --- | --- | --- |
| SOX4 | TGGTGGTTGTAGGAAGGTGT | CACCGTGTCCAACTCTGTCT |
| BMI1 | GCCCTTCATCCTCCTACC | GGTGTCCGTCAGGAAGTC |
| YBX1 | ACCTGGAAGGAGAGTGTGGA | GGGTGGGTGAGATCAGTG |
| SKP2 | GGTCTGATGGACCCAGAAGT | CCGCTGAACTCCAACTGATG |
| LTBP1 | AGGACTACGGGCTGTGGA | AGTGTGGCTGTCGATGATGG |
| PTK2 | GGGACCCCATGACTTCTCTC | GCCATTCCTTTCCCTTTCTC |
| RUNX1 | GTGGAGAGCTGGGAAACTGT | GTGGTGGTGGTGGTGGTTAG |
| SPK2 | CTGCCACCTGTGGTGTGTT | GGGACACCTTCAGGAGTGTG |
| DDX5 | CCCAAGGAGGAGAGGAGA | GTCCCGCTGTCTGAACTG |
| SLC30A7 | GCAGCAGGATGAGAAGAGGA | AGGGTCCAGGTGAGGAGTAA |
| ZNF24 | TGTGGGTGGTGTGCTGTGT | GGAGATGCAGAGCAGGAGAG |
| VEGF-A | ATGGAGATGAGCTTCCTAC | TTACAGTTCGTGTTTGGTGC |
| β-actin | AGAGCTACGAGCTGCCTGAC | AGCACTGTGTTGGCGTACAG |

**Western blotting**

Total protein was extracted using RIPA lysis buffer (Beyotime, Shanghai, China) containing 1% PMSF (Beyotime) and 1% phosphatase inhibitor (Solarbio, Beijing, China). The protein concentration was determined using a BCA assay kit (Beyotime). After denaturation, proteins were separated using 10% SDS-PAGE and then transferred to PVDF membranes (Millipore, Bedford, MA, USA). Primary antibodies of SOX4 (1:1000), Bmi1 (1:1000), E-cadherin (1:1000), N-cadherin (1:1000), Snail (1:1000), ZNF24 (1:2000), VEGF-A (1:1000), H2Aub (1:1000), Histone H3 (1:1000), or β-actin (1:1000) were incubated overnight at 4°C. Then, the secondary antibodies (1:5000) were applied for 1 hour. The protein bands were visualized using enhanced chemiluminescence (Millipore, Bedford, MA, USA).

**Transfection and stable cell lines**

To enhance the expression level of protein, the human SOX4, BMI1, or ZNF24 gene was PCR-amplified from cDNA and cloned into pcDNA3.1 plasmid (GeneChem, Shanghai, China). To silence the expression of SOX4, BMI1, or ZNF24, short hairpin RNA (shRNA) was transfected into the GV493 plasmid (GeneChem). H1299 or Calu-3 cells were plated at 2×10^6^ cells per 6-well plate and transfected with 10 µg of the indicated plasmid using lentiviral transfection. Stable cell lines were selected with media containing 2 μg/ml of puromycin for 14 days. The efficiency levels of knockdown and overexpression were verified by western blotting and qPCR.

The target sequences for shRNA were as follows:

| **Genes** | **The target sequence of shRNAs (5'- 3')** |
| --- | --- |
| shSOX4 | GGAAGGAGAGGACUCAACA |
| shBMI1-1 | GGAGGAGGAGAGAAGAGGA |
| shBMI1-2 | GCUGAAGGAGGAGAGAGGA |
| shZNF24-1 | GAAGAGGAGGAGAAGAGAA |
| shZNF24-2 | GGAAGAGGAGGAGAAGAGA |
| Scramble | UUCUCCGAACGUGUCACGU |

**Conditioned medium preparation**

H1299 and Calu-3 cells were incubated in DMEM medium containing 10% FBS until the confluence became 30%-50%. The medium was collected and subjected to centrifugation at 1000×g to discard the pellets. The supernatant was filtered with a low-protein-binding filter (0.2µm) (Millipore) and concentrated ten-fold using an Amicon Ultra filter (Millipore) at 4000×g.

**CCK-8 assay**

H1299 and Calu-3 cells were seeded into a 96-well plate (5×10^3^ cells/well) and incubated continuously for 6 days. Every 24 hours, 10 µL of CCK-8 reagent (Dojindo, Kumamoto, Japan) was added, followed by a 40-minute incubation at 37°C. Subsequently, the absorbance values at 450 nm were measured using a spectrophotometer (Molecular Devices, France) to assess the proliferation level of the cells.

Similarly, HUVEC cells were seeded into a 96-well plate (5×10^3^ cells/well) and incubated with different conditioned medium continuously for 80 hours. The absorbance values at 450 nm were measured every 20 hours using a spectrophotometer.

**Transwell assay**

The 8.0 μm pore Transwell chamber was purchased from BD Biosciences (Franklin Lakes, NJ, USA). The upper chamber was left uncoated with Matrigel for the migration assay while pre-coating with 50 μL of Matrigel (BD Biosciences) was applied for the invasion assay. H1299 and Calu-3 cells were serum-starved for 6 hours before the experiment. A total of 5×10^4^ cells were suspended in a serum-free culture medium and plated into the upper chamber. The lower chambers contained media with 10% FBS. After 16 hours of incubation, cells adhering to the bottoms of chambers were fixed with 4% methanol and stained with 0.1% crystal violet (Sigma-Aldrich) for 30 minutes. Three random fields were chosen under the microscope, and the numbers of migrated or invaded cells were quantified using Image J software (National Institutes of Health, NY, USA).

**Wound healing assay**

HUVEC cells were seeded in 6-well plates (2×10^5^ cells/well) and cultured at 37 °C. After attachment, a wound was created in the cell monolayer using a sterile pipette tip. Cells were washed twice with cold PBS, and the initial wound size was measured using a microscope. Then, cells were cultured with serum-free medium for 24 hours at 37 °C, and the wound size was measured again. The wound closure percentage=(1 − [final wound size/initial wound size])×100.

**Vascular tube formation assay**

The vascular tube formation assay was conducted using an in vitro Angiogenesis Assay Kit (Thermo Fisher) following the manufacturer's instructions. First, 96-well tissue culture plates were coated with growth factor-reduced Matrigel (BD Biosciences). HUVEC cells were starved in serum-free medium for 6 hours and then seeded into the 96-well tissue culture plates at a concentration of 1×10^4^ cells per well. The plates were then incubated at 37°C for 6 hours. The cells were visualized using a light microscope, and pictures of the capillary network were taken. The tube lengths were counted using Scion Image software (BD Biosciences).

**Chromatin Immunoprecipitation-qPCR**

Chromatin immunoprecipitation (ChIP) was conducted using an enzymatic ChIP kit (Cell Signaling Technology, Cat. No. 9003) according to the manufacturer's instructions. Briefly, H1299 and Calu-3 cells were sequentially treated with DTBP solution (Sigma-Aldrich Cat. No. D2338) and formaldehyde and harvested with a cell scraper. Then, the cell pellet was lysed with ChIP lysis buffer and sonicated to generate fragmented chromatin samples. Chromatin samples were incubated with anti-SOX4 antibodies (Abcam, Cat. No. Ab86809) and anti-Ubiquityl-Histone H2A (Cell Signaling Technology, Cat. No. D27C4). Rabbit IgG (Cell Signaling Technology, Cat. No. 2729) was used as the negative control. A non-immunoprecipitated sample (2%) was used as the input control. The purified DNA was then detected by qPCR. The primer sequences for ChIP-qPCR are listed in the following table:

| **Target genes** | **Forward primer (5'- 3')** | **Reverse primer (5'- 3')** |
| --- | --- | --- |
| BMI1 | AACCTGTGCTCCACCTCTC | TCCCTTCCCTTCCTCCCT |
| PTK2 | AGAGTGAGGAGCAGGAAGGA | GCTGCAGTGGTGGTGTGT |
| ZNF24 | AGGCTGTCTCTGCTTTGCAT | CAGGTCACCCACCTTTCCAT |

**Dual-luciferase reporter assay**

The BMI1 promoter region sequence was queried using the NCBI database. Specifically, after identifying the gene location of BMI1, the upstream 2000bp was taken as the predicted promoter region sequence. The binding sites of SOX4 to the predicted BMI1 promoter region were identified using the transcription factor prediction software (JASPAR). In the prediction results, we screened out potential binding sites based on the conditions of being located on the same nucleotide strand as the BMI1 gene, having an absolute score greater than 5, and a relative score greater than 0.8 (Supplementary Table 4). We then further screened these using ChIP-qPCR (Supplementary Table 5), ultimately identifying two potential binding sequences.

For these two sites in the BMI1 promoter region, we constructed mutations and conducted dual-luciferase experiments. Specifically, the human promoter region generated by PCR amplification was cloned into the KpnI/HindIII site of the PGL3-basic dual-luciferase reporter plasmid to generate the BMI1 reporter gene. Subsequently, stably transfected H1299 cells (5×10^4^ cells per well) were seeded into 24-well plates and transfected with the reporter plasmid and pRL-TK Renilla plasmid using Lipofectamine 3000 (Thermo Fisher). After 48 hours, luciferase activity and Renilla signal were detected using the Dual-Luciferase Reporter Assay System (Promega, Madison, WI, USA). Renilla luciferase was used as an internal control to eliminate the influence of transfection efficiency. The activity of the reporter gene was determined by normalizing firefly luciferase activity to Renilla luciferase activity.

**ELISA assay**

The Human VEGF-A ELISA kit (Sigma-Aldrich, Cat. No. RAB0507) was used to measure the concentration of VEGF-A in cell culture medium and serum according to the manufacturer's instructions. Before detection, the serum or culture medium was also concentrated at 1000×g to remove the pellets. Prepare standard samples and dilute them in a gradient. For ELISA detection, 100 µL of standard or sample was added to the appropriate wells and incubated for 2 hours at room temperature. After washing, 200 µL of VEGF-A conjugate was added and incubated for 2 hours at room temperature. Substrate solution was added for 30 minutes at room temperature. After adding 50 µL of stop solution, the ELISA absorbance values could be read on a BIOTEK spectrophotometer (Vermont, USA) at 450 nm.

**Bioinformatic analysis**

The expression levels of SOX4 in NSCLC were analyzed using the UALCAN website (https://ualcan.path.uab.edu). The analysis includes the LUAD (lung adenocarcinoma) dataset and LUSC (lung squamous cell carcinoma) dataset, along with the respective results from normal tissues serving as controls[1, 2].

The ChIP-seq datasets from fetal lung fibroblasts with the SOX4 ChIP antibody were downloaded from the GEO database (GSM1970164, GSM1970165). Utilizing these datasets, we intersected them with EMT-associated genes, aiming to identify potential downstream targets of SOX4. Similarly, to identify potential downstream targets of BMI1, we downloaded the ChIP-seq datasets from the GEO database (GSM2828726, GSM1612057, and GSM1138595). Subsequently, we intersected these datasets with RNA sequencing results (GSE163175).

**Reference**

[1] D.S. Chandrashekar, B. Bashel, S.A.H. Balasubramanya, C.J. Creighton, I. Ponce-Rodriguez, B. Chakravarthi, S. Varambally, UALCAN: A Portal for Facilitating Tumor Subgroup Gene Expression and Survival Analyses, Neoplasia, 19 (2017) 649-658.

[2] D.S. Chandrashekar, S.K. Karthikeyan, P.K. Korla, H. Patel, A.R. Shovon, M. Athar, G.J. Netto, Z.S. Qin, S. Kumar, U. Manne, C.J. Creighton, S. Varambally, UALCAN: An update to the integrated cancer data analysis platform, Neoplasia, 25 (2022) 18-27.

**Supplementary Figures**


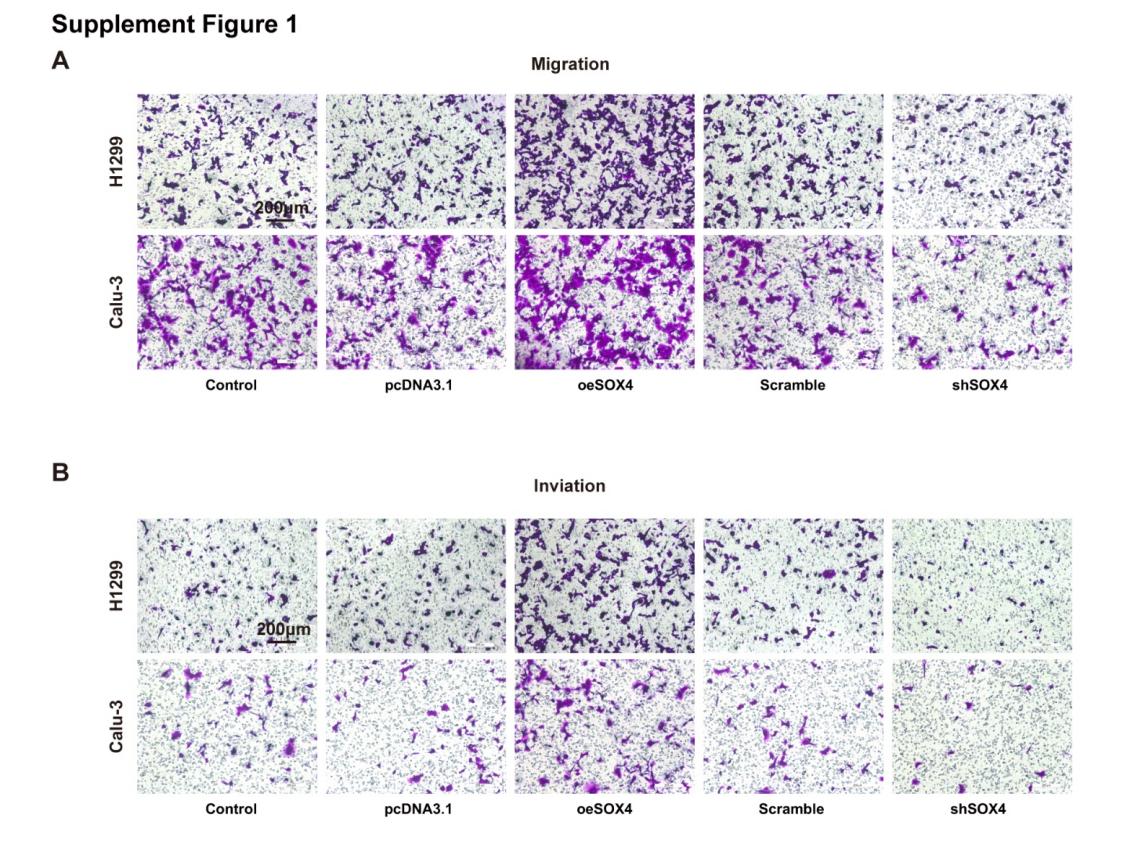


**Supplementary Figure 1.**

1. Representative images of the migration assay are in Figure 2F. Scale bars, 200 μm
2. Representative images of the invasion assay are in Figure 2G. Scale bars, 200 μm


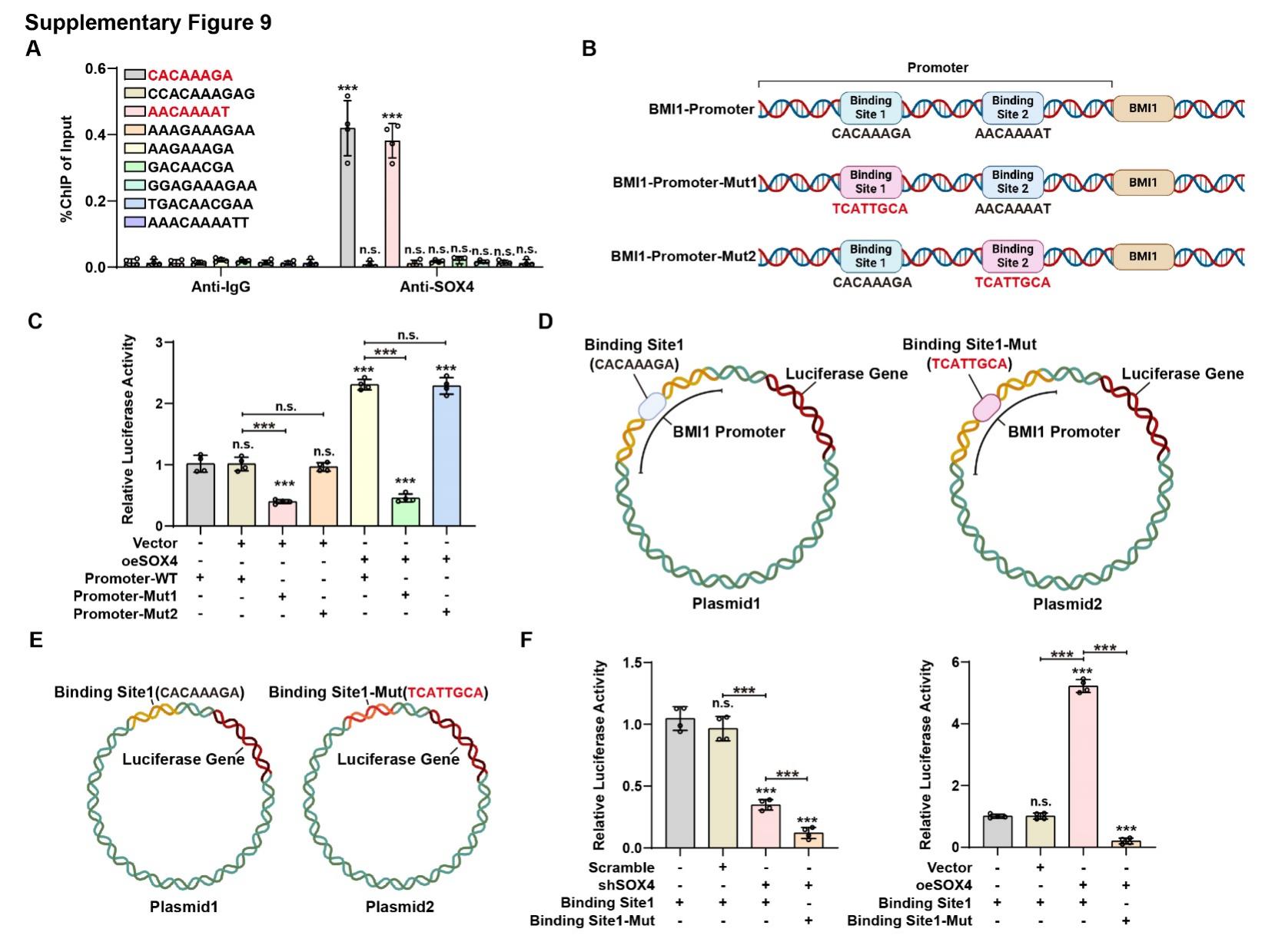


**Supplementary Figure 2.**

1. Primers were designed for regions within the BMI1 promoter that were predicted by JASPAR to bind SOX4, followed by SOX4 ChIP-qPCR analysis. Two preferred binding sequences for SOX4 were identified in H1299 cells.
2. Schematic representation of the mutant plasmids constructed with the preferred SOX4 binding sequences in the BMI1 promoter region. These plasmids are used for subsequent luciferase reporter assays. (C) Dual-luciferase assay using plasmids constructed in (B) to further investigate the sequences in the BMI1 promoter region where SOX4 can bind and exert its function.

(D) The two specially constructed plasmids used in the dual-luciferase assay are shown in Figure 3G. The plasmid on the left contains the full BMI1 promoter sequence, while the plasmid on the right contains the BMI1 promoter sequence with mutations in the SOX4 binding regions based on the results from the experiment (C).

(E) Schematic representation of the dual-luciferase plasmids constructed with only the SOX4 binding regions and their mutated sequences, used for subsequent experiments.

(F) Dual-luciferase assay using the plasmids constructed in (E), demonstrating that SOX4 binding to its preferred sequences can activate promoter function.

n.s. represents not significant; ** and *** represents *P*<0.01 and 0.001 respectively. Data were analyzed with paired *t*-test (A, C, and F). Data were from at least 3 independent experiments and shown as mean ± S.E.M.


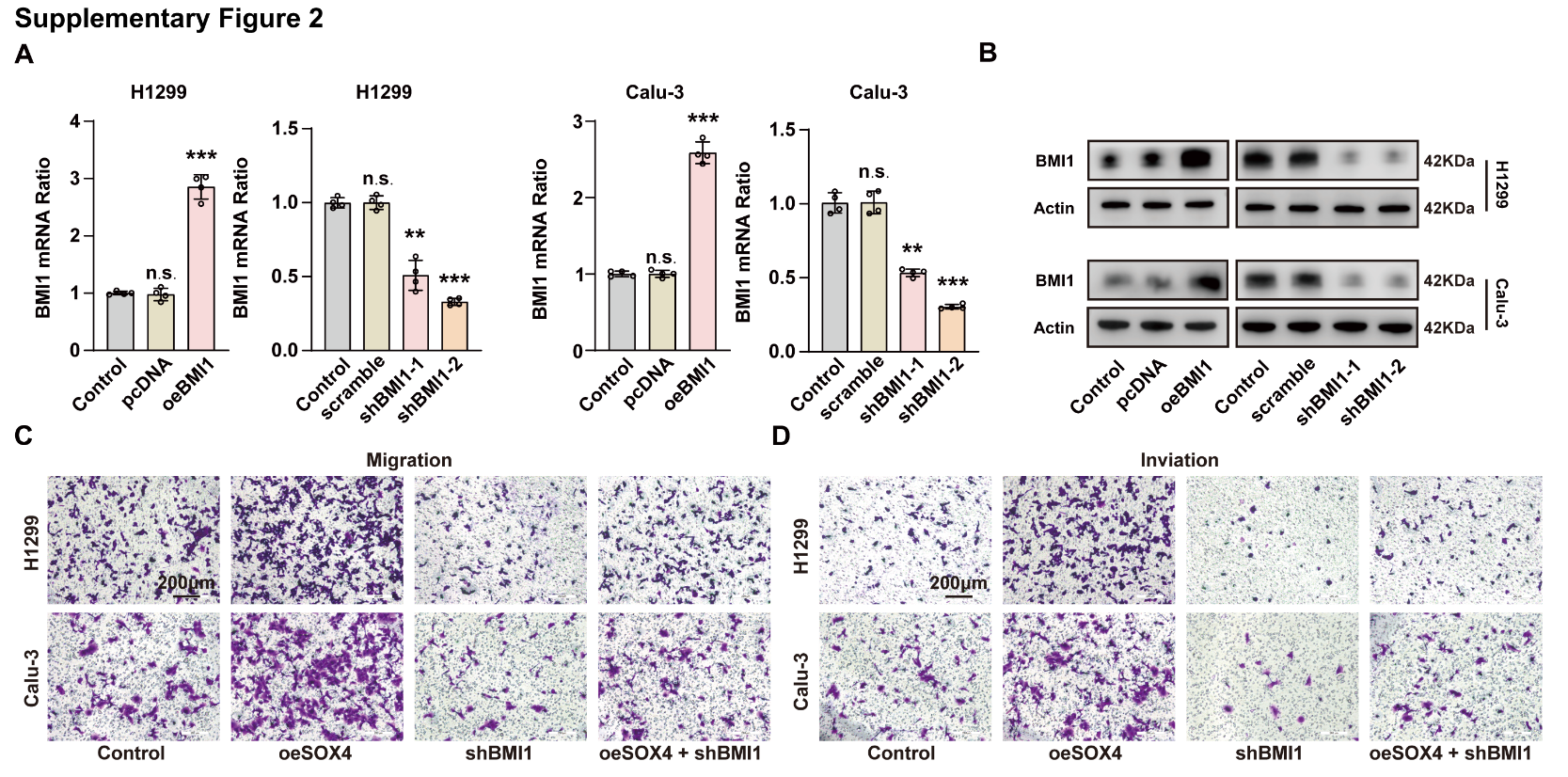


**Supplementary Figure 3.**

(A, B) Successful knockdown and overexpression of BMI1 in H1299 and Calu-3 cells were confirmed by qPCR (A) and western blot (B).

(C) Representative images of the migration assay are in Figure 4D. Scale bars, 200μm

(D) Representative images of the invasion assay are in Figure 4E. Scale bars, 200μm

n.s. represents not significant; ** and *** represents *P*<0.01 and 0.001 respectively. Data were analyzed with paired *t*-test (A). Data were from at least 3 independent experiments and shown as mean ± S.E.M.


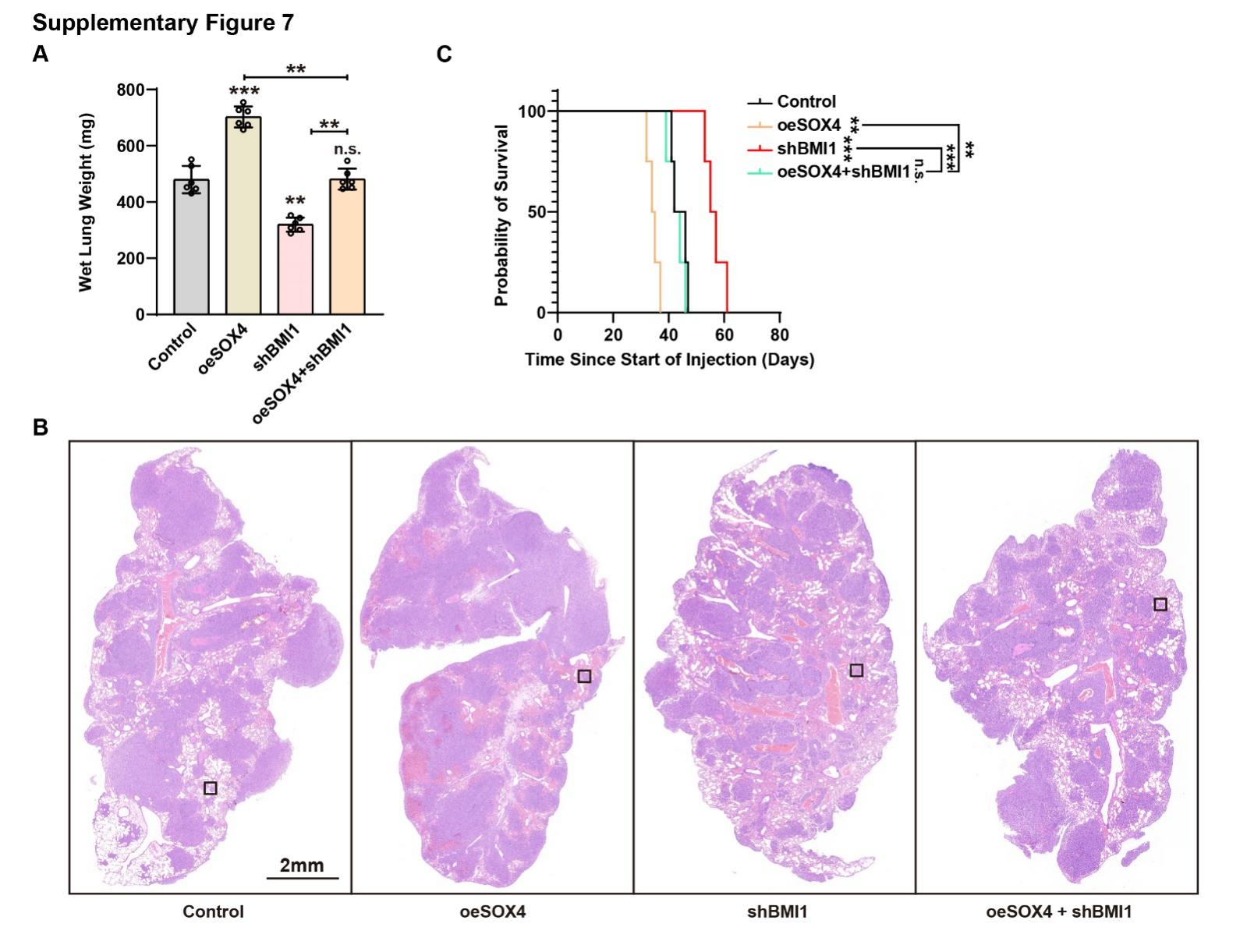


**Supplementary Figure 4.**

1. Orthotopic model in nude mice was established with stable SOX4 overexpression, BMI1 silencing, and simultaneous stable SOX4 overexpression with BMI1 silencing in H1299 cells. The wet lung weight in the orthotopic model was measured.
2. Representative IHC images of the largest cross-section of the right lung lobe. Scale bars, 2 mm
3. Survival curve of mice since the start of H1299 cell injection.

n.s. represents not significant; ** and *** represents *P*<0.01 and 0.001 respectively. Data were analyzed with paired *t*-test (A).


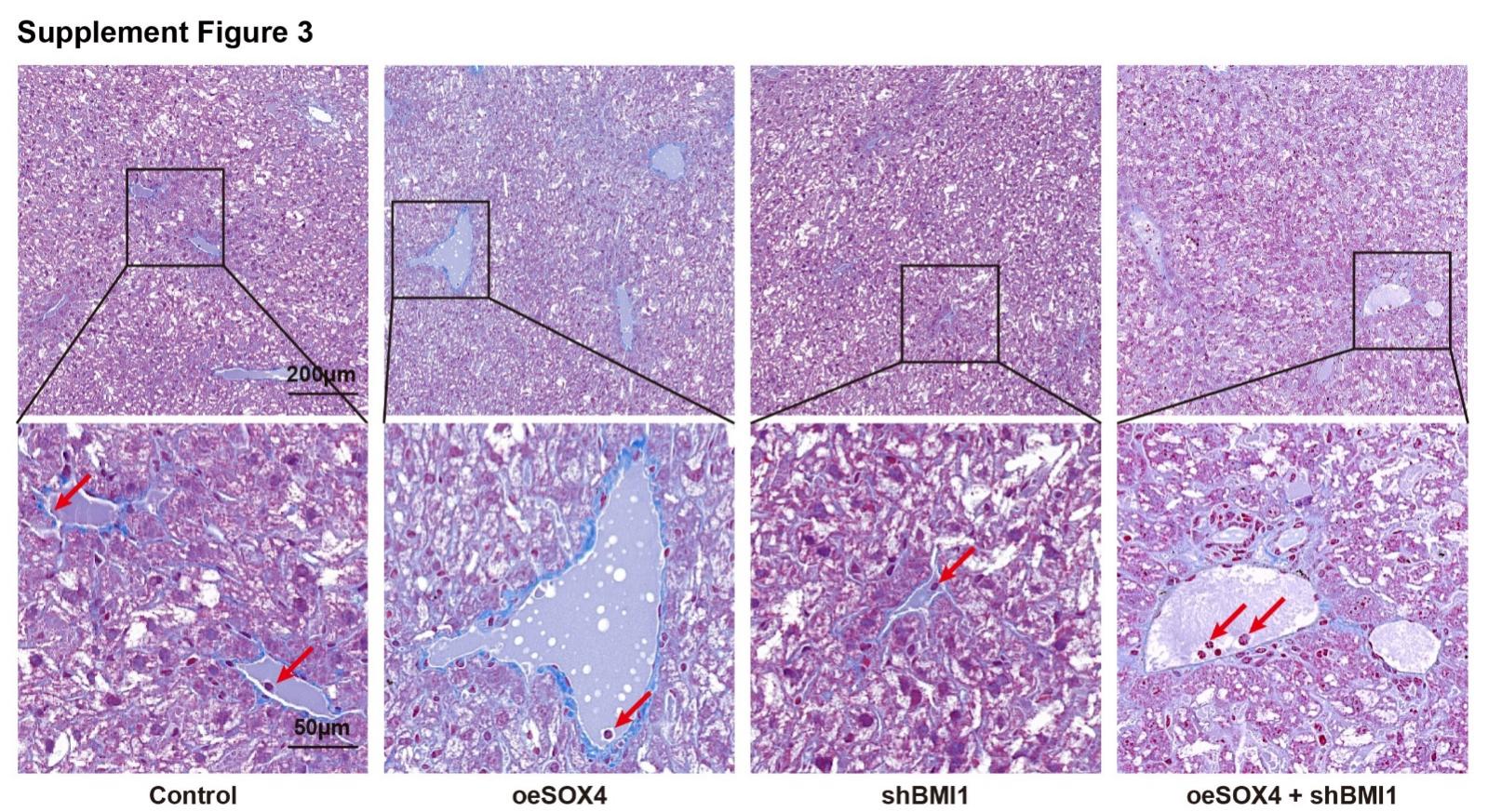


**Supplementary Figure 5.**

Representative Masson's staining images demonstrate increased angiogenesis in tumors when SOX4 is overexpressed, a trend that is eliminated upon BMI1 knockdown. upper: scale bars, 200 μm; lower: scale bars, 50 μm


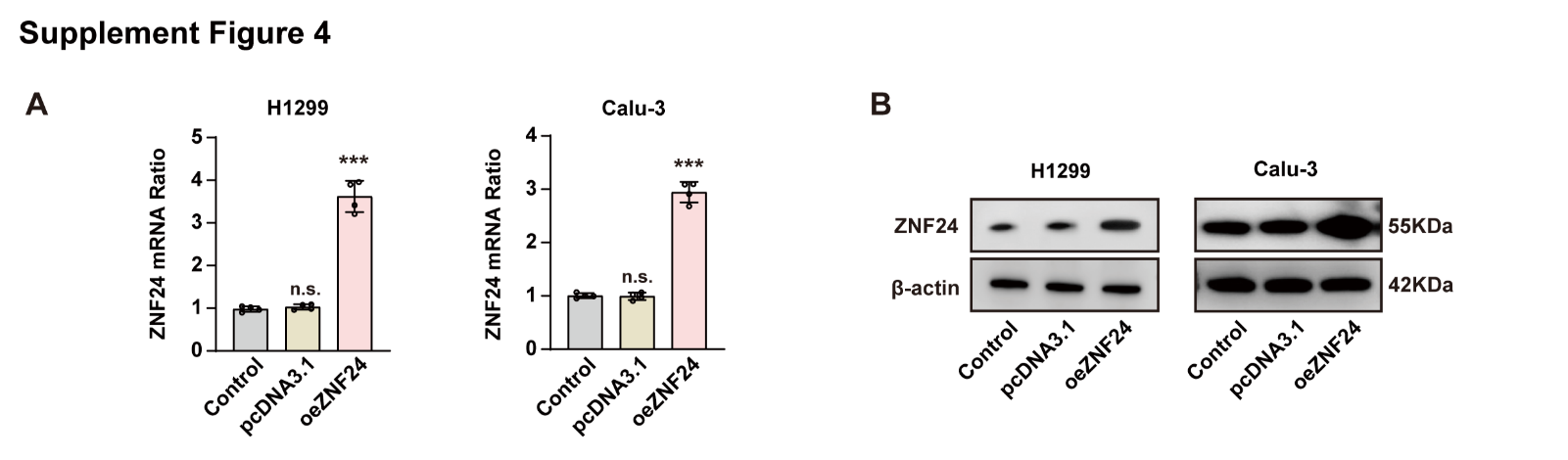


**Supplementary Figure 6.** (A, B) Successful overexpression of ZNF24 in H1299 and Calu-3 cells was confirmed by qPCR(A) and western blot (B).

n.s. represents not significant; ** and *** represents *P*<0.01 and 0.001 respectively. Data were analyzed with paired *t*-test (A). Data were from at least 3 independent experiments and shown as mean ± S.E.M.


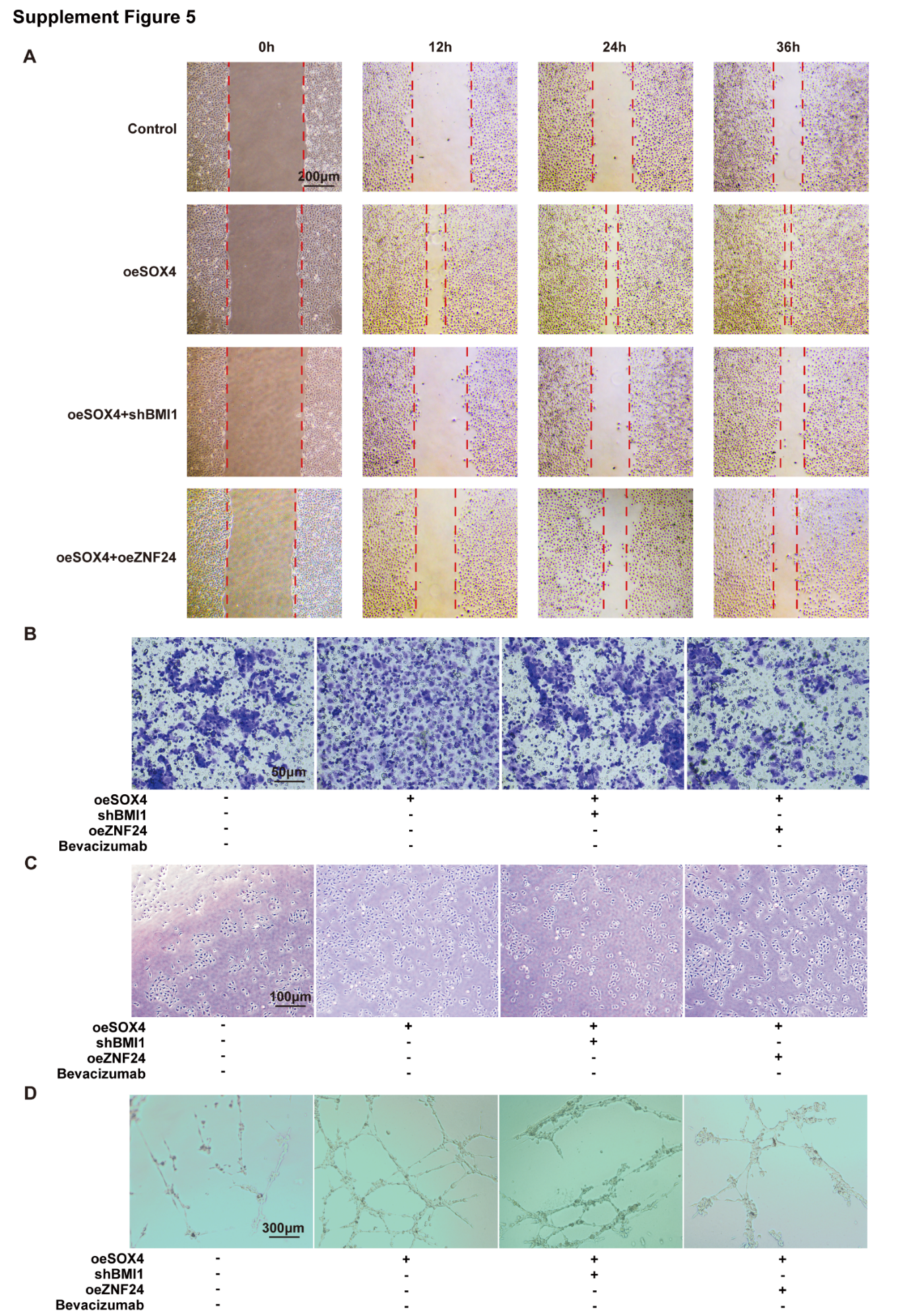


**Supplementary Figure 7.**

1. Representative images of the wound healing assay in Figure 5K. Scale bars, 200μm.
2. Representative images of the migration assay in Figure 5L. Scale bars, 50μm.
3. Representative images of the proliferation assay in Figure 5M. Scale bars, 100μm.
4. Representative images of the angiogenesis assay in Figure 5N. Scale bars, 300μm.


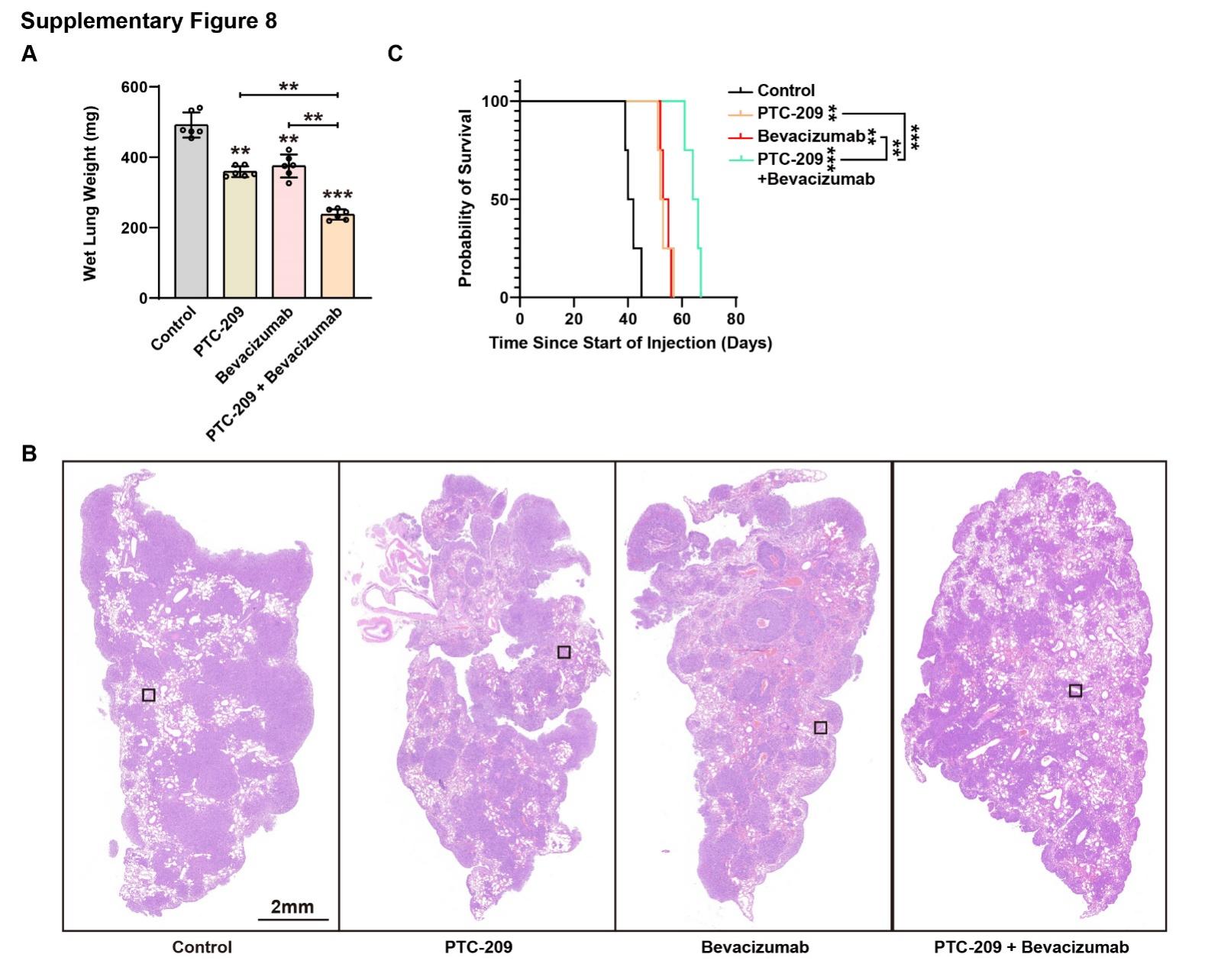


**Supplementary Figure 8.**

1. Orthotopic model in nude mice was established with H1299 cells. After two weeks, PTC-209 (60 mg/kg/d, s.c.) or Bevacizumab (5 mg/kg/3d, i.v.) was used. The wet lung weight in the orthotopic model was measured.
2. Representative IHC images of the largest cross-section of the right lung lobe. Scale bars, 2 mm
3. Survival curve of mice since the start of H1299 cell injection.

n.s. represents not significant; ** and *** represents *P*<0.01 and 0.001 respectively. Data were analyzed with paired *t*-test (A).


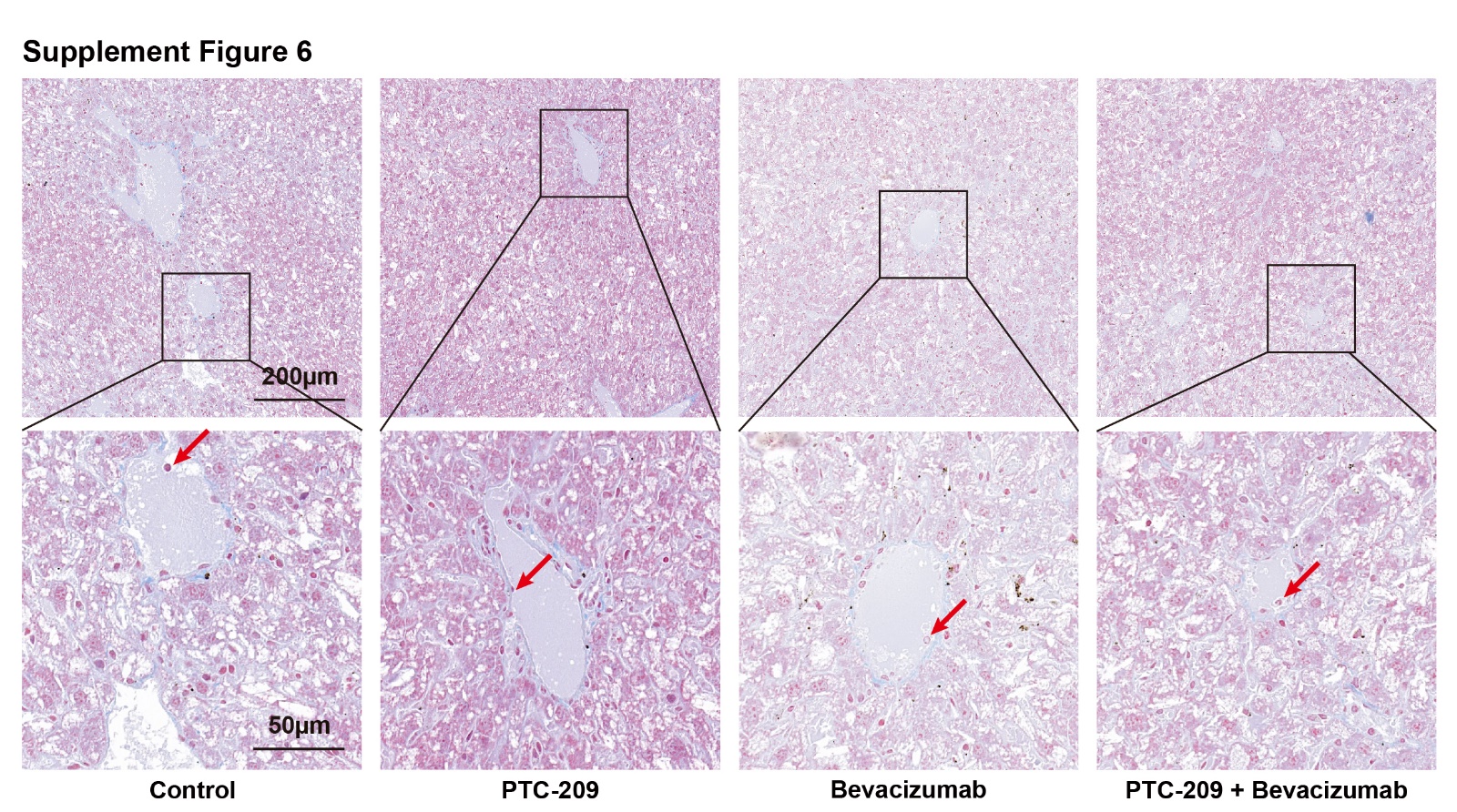


**Supplementary Figure 9.**

The representative image of Masson's staining demonstrates that the combination of the BMI1-specific inhibitor PTC209 with bevacizumab significantly reduces angiogenesis in lung cancer tissue. In the tumor tissues of the combined treatment group with PTC-209 and bevacizumab, a reduction in the number of blood vessels and a decrease in the diameter of newly formed blood vessels were observed under the microscope. upper: scale bars, 200 μm; lower: scale bars, 50 μm

**Supplementary Tables**

**Table 1. The expression of SOX4 in NSCLC.**

| **Characteristics** | **Validated cohort (n=93)** | |  |
| --- | --- | --- | --- |
|  | SOX4^low^  (n=62) | SOX4^high^  (n=31) | |
| **Age (years)**  <65  ≥65 |  |  | |
|  | 36 | 18 | |
|  | 26 | 13 | |
| **Gender**  female  male |  |  | |
|  | 21 | 13 | |
|  | 41 | 18 | |
| **Tumor size**  <3.0cm  ≥3.0cm |  |  | |
|  | 18 | 12 | |
|  | 34 | 19 | |
| **Differentiation**  Well+Moderate  Poor |  |  | |
|  | 37 | 9 | |
|  | 25 | 22 | |
| **T stage**  T1+T2  T3+T4 |  |  | |
|  | 41 | 13 | |
|  | 21 | 18 | |
| **N stage**  N0  N1+N2 |  |  | |
|  | 37 | 17 | |
|  | 25 | 14 | |
| **M stage**  M0  M1 |  |  | |
|  | 60 | 27 | |
|  | 1 | 5 | |
| **TNM stage**  I+II  III+IV |  |  | |
|  | 15 | 7 | |
|  | 47 | 24 | |

**Abbreviations:** NSCLC: non-small cell lung cancer.

**Table 2. The expression of BMI1 in NSCLC.**

| **Characteristics** | **Validated cohort (n=93)** | |  |
| --- | --- | --- | --- |
|  | BMI1^low^  (n=53) | BMI1^high^  (n=40) | |
| **Age (years)**  <65  ≥65 |  |  | |
|  | 32 | 22 | |
|  | 21 | 18 | |
| **Gender**  female  male |  |  | |
|  | 20 | 14 | |
|  | 33 | 26 | |
| **Tumor size**  <3.0cm  ≥3.0cm |  |  | |
|  | 26 | 14 | |
|  | 27 | 26 | |
| **Differentiation**  Well+Moderate  Poor |  |  | |
|  | 34 | 12 | |
|  | 19 | 28 | |
| **T stage**  T1+T2  T3+T4 |  |  | |
|  | 31 | 23 | |
|  | 22 | 17 | |
| **N stage**  N0  N1+N2 |  |  | |
|  | 41 | 21 | |
|  | 12 | 19 | |
| **M stage**  M0  M1 |  |  | |
|  | 51 | 36 | |
|  | 2 | 4 | |
| **TNM stage**  I+II  III+IV |  |  | |
|  | 13 | 9 | |
|  | 40 | 31 | |

**Abbreviations:** NSCLC: non-small cell lung cancer.

**Table 3. The prognostic significance of clinicopathological characteristics in NSCLC.**

| **Characteristics** | | **3-year OS** | ***p^a^*** | **HR** | **95%CI** | ***p^b^*** |
| --- | --- | --- | --- | --- | --- | --- |
| **Age (years)** | <65 | 38.0 | 0.588 |  |  |  |
|  | ≥65 | 25.6 |  |  |  |  |
| **Gender** | male | 33.3 | 0.875 |  |  |  |
|  | female | 35.0 |  |  |  |  |
| **Tumor size** | <3.0cm | 50.1 | **0.007** | 1 |  | 0.169 |
|  | ≥3.0cm | 14.4 |  | 1.330 | 0.62-2.56 |  |
| **Differentiation** | Well+Moderate | 24.2 | 0.091 |  |  |  |
|  | Poor | 30.8 |  |  |  |  |
| **T stage** | T1+T2 | 43.9 | **0.015** | 1 |  | 0.175 |
|  | T3+T4 | 22.5 |  | 0.908 | 0.51-1.76 |  |
| **N stage** | N0 | 37.5 | **0.021** | 1 |  | 0.578 |
|  | N1+N2 | 20.3 |  | 0.948 | 0.48-1.77 |  |
| **M stage** | M0 | 43.1 | **0.039** | 1 |  | **0.007** |
|  | M1 | 0.00 |  | 2.703 | 1.07-5.02 |  |
| **TNM stage** | I+II | 41.4 | **0.012** |  |  |  |
|  | III+IV | 20.6 |  |  |  |  |
| **SOX4** | Low | 47.5 | **0.001** | 1 |  | **0.001** |
|  | High | 14.5 |  | 3.497 | 1.60-6.17 |  |
| **BMI1** | Low | 42.9 | **0.001** | 1 |  | **0.001** |
|  | High | 17.0 |  | 1.615 | 1.09-2.21 |  |
| **SOX4+BMI1** | BMI1^high^+Foxn2^low^ | 16.7 | **0.001** |  |  |  |
|  | Others | 41.2 |  |  |  |  |

**Abbreviations:** NSCLC: non-small cell lung cancer; OS: overall survival; HR: hazard ratio; CI: confidence interval; *p^a^* calculated by log-rank test; *p^b^* calculated by Cox-regression hazard model.

**Table 4. JASPAR predicts the SOX4 binding sequence in the BMI1 promoter region**

| **Score** | **Relative Score** | **Sequence ID** | **Start** | **End** | **Strand** | **Predicted Sequence** |
| --- | --- | --- | --- | --- | --- | --- |
| 13.267487 | 1.000000009 | NC_000010.11:22319099-22321099 | 558 | 565 | - | AACAAAGG |
| 11.280262 | 0.942551244 | NC_000010.11:22319099-22321099 | 557 | 566 | - | TAACAAAGGT |
| **10.008083** | **0.937968503** | **NC_000010.11:22319099-22321099** | **1203** | **1210** | **+** | **CACAAAGA** |
| **9.587727** | **0.912262617** | **NC_000010.11:22319099-22321099** | **1202** | **1211** | **+** | **CCACAAAGAG** |
| 6.9997077 | 0.865948939 | NC_000010.11:22319099-22321099 | 1218 | 1227 | - | AAACAAAATA |
| 6.903564 | 0.864228407 | NC_000010.11:22319099-22321099 | 1249 | 1258 | - | ACACAAAGTA |
| **6.6625643** | **0.874298079** | **NC_000010.11:22319099-22321099** | **221** | **228** | **+** | **AACAAAAT** |
| 6.6625643 | 0.874298079 | NC_000010.11:22319099-22321099 | 1219 | 1226 | - | AACAAAAT |
| 6.56635 | 0.872466971 | NC_000010.11:22319099-22321099 | 1250 | 1257 | - | CACAAAGT |
| **6.279316** | **0.853057228** | **NC_000010.11:22319099-22321099** | **1711** | **1720** | **+** | **AAAGAAAGAA** |
| 6.0710583 | 0.8630408 | NC_000010.11:22319099-22321099 | 15 | 22 | - | GACAAATG |
| 6.024422 | 0.848495797 | NC_000010.11:22319099-22321099 | 14 | 23 | - | TGACAAATGG |
| **5.92971** | **0.860350722** | **NC_000010.11:22319099-22321099** | **1712** | **1719** | **+** | **AAGAAAGA** |
| 5.92971 | 0.860350722 | NC_000010.11:22319099-22321099 | 770 | 777 | - | AAGAAAGA |
| 5.9252243 | 0.84672061 | NC_000010.11:22319099-22321099 | 969 | 978 | - | GCACAAACGG |
| **5.7803364** | **0.857507911** | **NC_000010.11:22319099-22321099** | **406** | **413** | **+** | **GACAACGA** |
| **5.6445913** | **0.841698566** | **NC_000010.11:22319099-22321099** | **1707** | **1716** | **+** | **GGAGAAAGAA** |
| **5.642425** | **0.8416598** | **NC_000010.11:22319099-22321099** | **405** | **414** | **+** | **TGACAACGAA** |
| 5.5581846 | 0.840152282 | NC_000010.11:22319099-22321099 | 1574 | 1583 | - | AAACAACAGC |
| 5.5185857 | 0.852526389 | NC_000010.11:22319099-22321099 | 1575 | 1582 | - | AACAACAG |
| **5.1583214** | **0.832996562** | **NC_000010.11:22319099-22321099** | **220** | **229** | **+** | **AAACAAAATT** |
| 4.922846 | 0.828782629 | NC_000010.11:22319099-22321099 | 818 | 827 | - | ATACAATACC |
| 4.8878345 | 0.840522215 | NC_000010.11:22319099-22321099 | 819 | 826 | - | TACAATAC |
| 4.688884 | 0.836735873 | NC_000010.11:22319099-22321099 | 970 | 977 | - | CACAAACG |
| 4.524753 | 0.821658593 | NC_000010.11:22319099-22321099 | 250 | 259 | - | AAGCAAAGAC |
| 4.4957895 | 0.833060989 | NC_000010.11:22319099-22321099 | 1346 | 1353 | - | GACAAACC |
| 4.49153 | 0.832979923 | NC_000010.11:22319099-22321099 | 1708 | 1715 | + | GAGAAAGA |
| 4.442168 | 0.832040493 | NC_000010.11:22319099-22321099 | 251 | 258 | - | AGCAAAGA |
| 4.362463 | 0.818754344 | NC_000010.11:22319099-22321099 | 677 | 686 | - | AAAAAAAGCG |
| 4.275932 | 0.817205833 | NC_000010.11:22319099-22321099 | 1191 | 1200 | - | AAACAACACC |

**Table 5. The primers for the SOX4 preferred binding sequence in the selected BMI1 promoter region.**

| **No.** | **Forward primer (5'- 3')** | **Reverse primer (5'- 3')** |
| --- | --- | --- |
| **1** | CACAAAGA | TCTTTGTG |
| **2** | CCACAAAGAG | CTCTTTGTGG |
| **3** | AACAAAAT | ATTTTGTT |
| **4** | AAAGAAAGAA | TTCTTTCTTT |
| **5** | AAGAAAGA | TCTTTCTT |
| **6** | GACAACGA | TCGTTGT |
| **7** | GGAGAAAGAA | TTCTTTCTCC |
| **8** | TGACAACGAA | TTCGTTGTC |
| **9** | AAACAAAATT | AATTTTGTT |
