# Supplementary material for: SOX4-BMI1 axis promotes non-small cell lung cancer progression and facilitates angiogenesis by suppressing ZNF24
Source: Cell Death Dis. 2024 Sep 30;15(9):698. doi: 10.1038/s41419-024-07075-w (PMC11442842; doi:10.1038/s41419-024-07075-w)

Figure 1C

SOX4

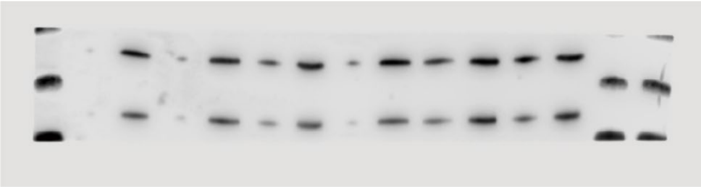

β-actin

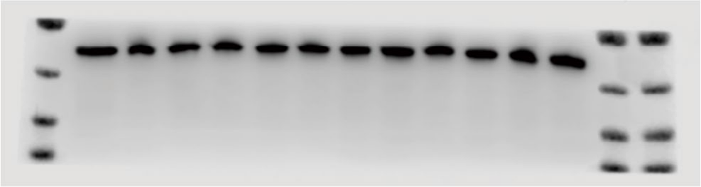

Figure 2B

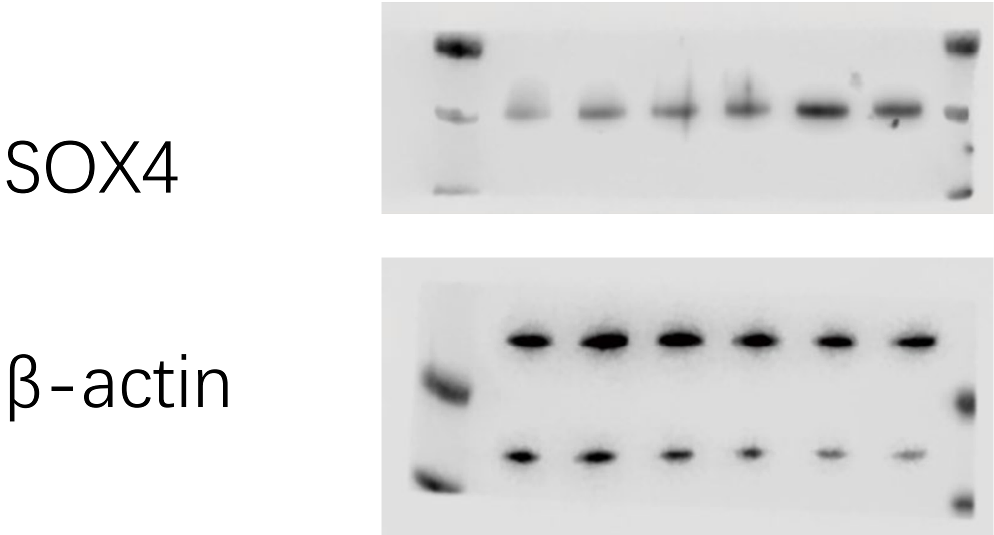

Figure 2D

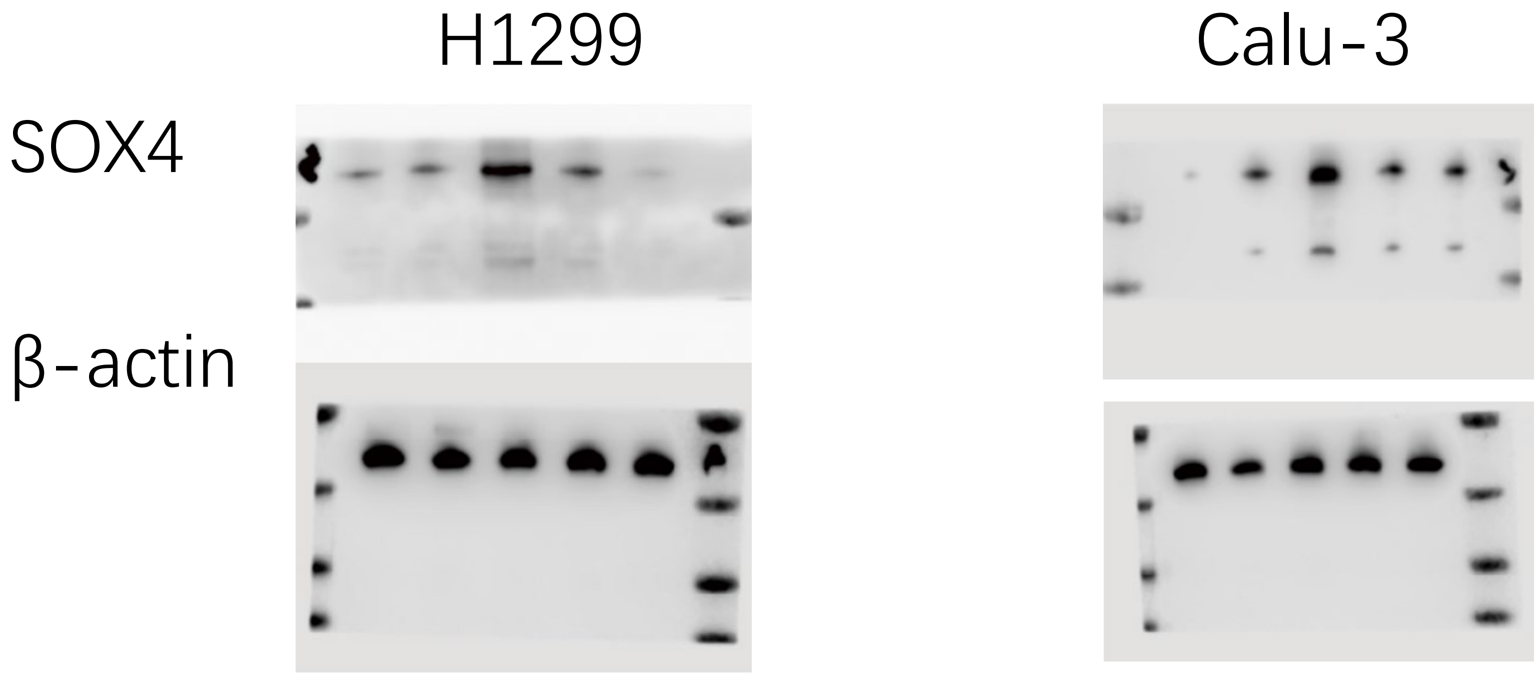

Figure 2H

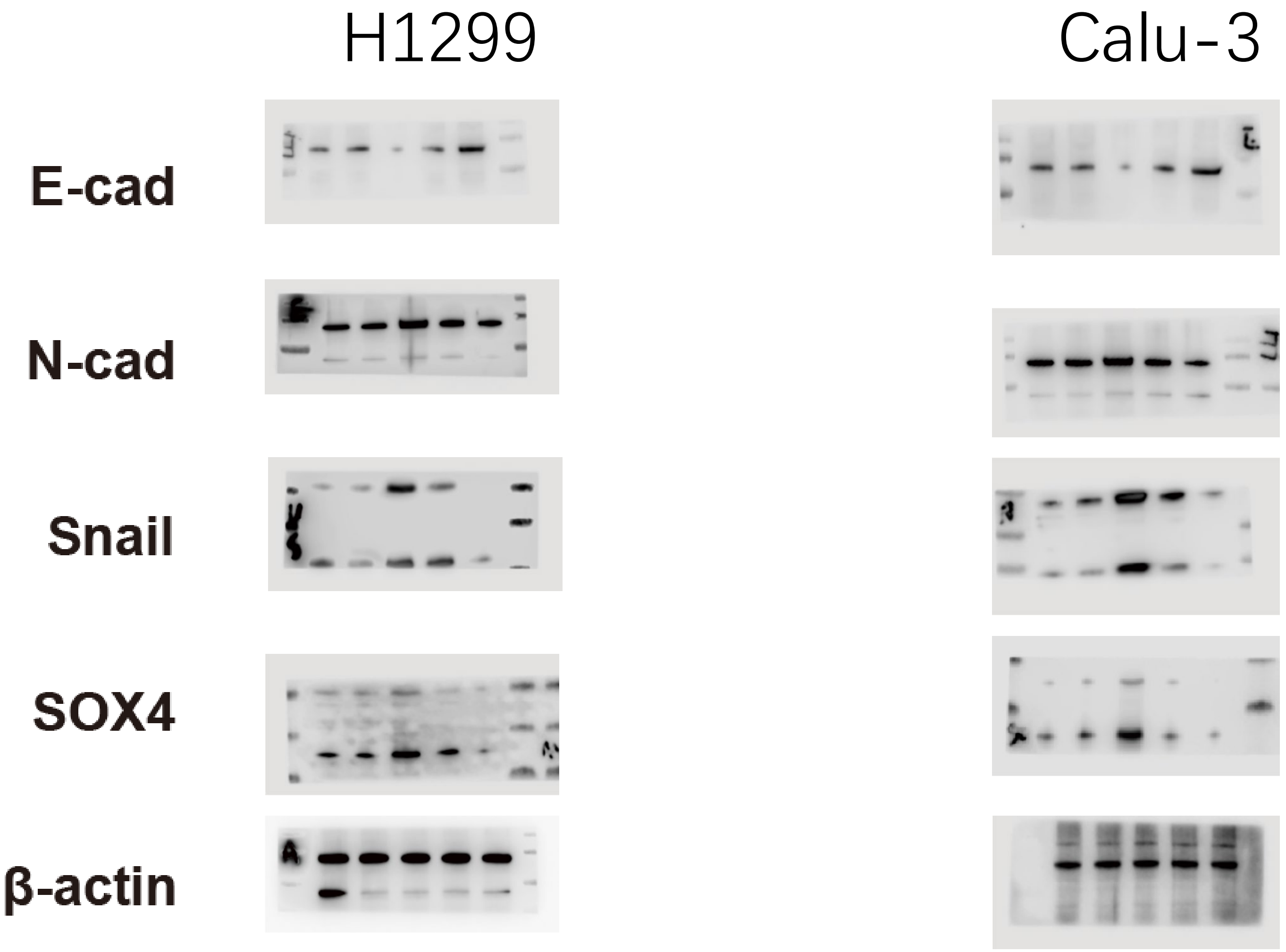

Figure 3H

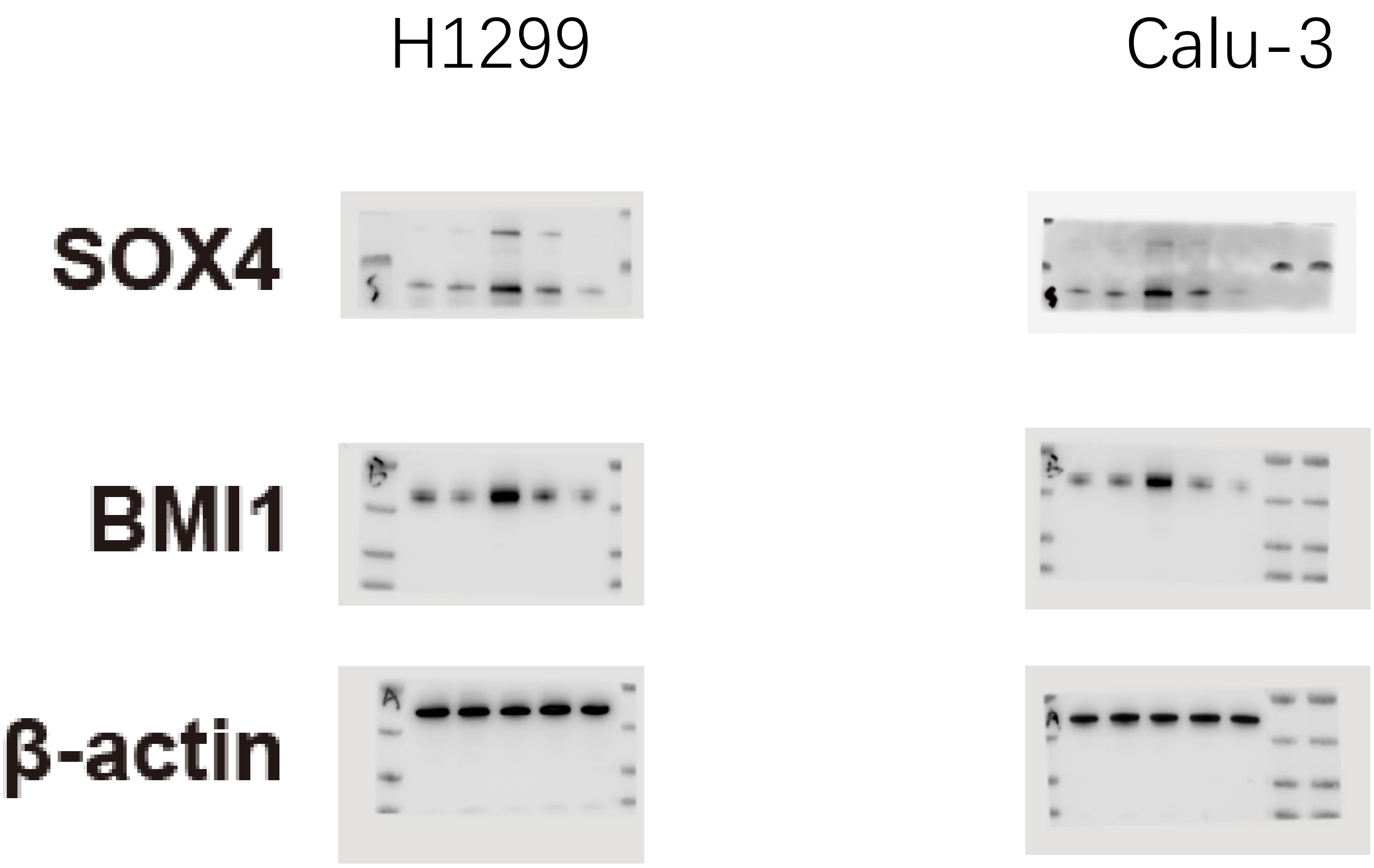

Figure 4B

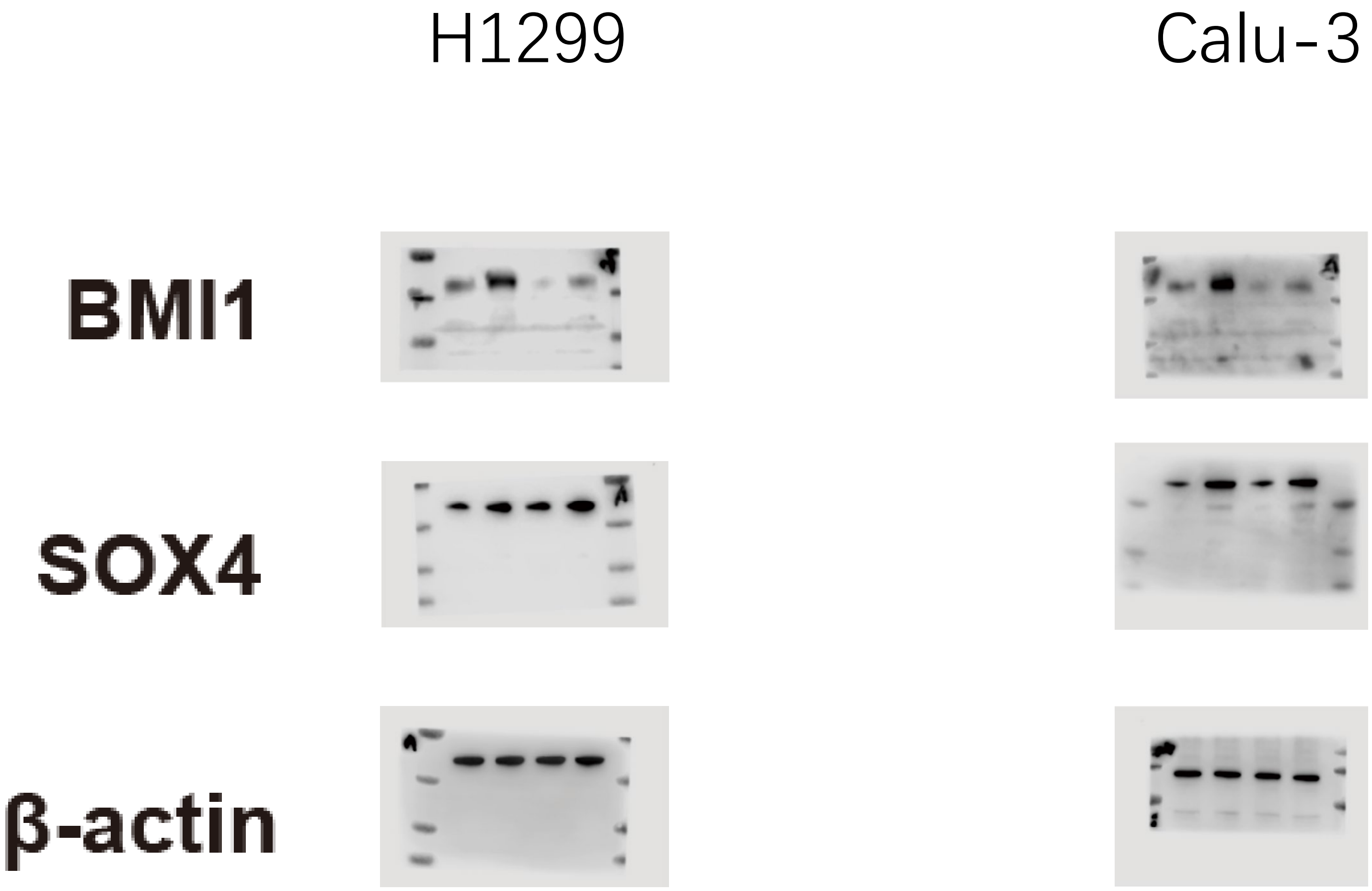

Figure 4F

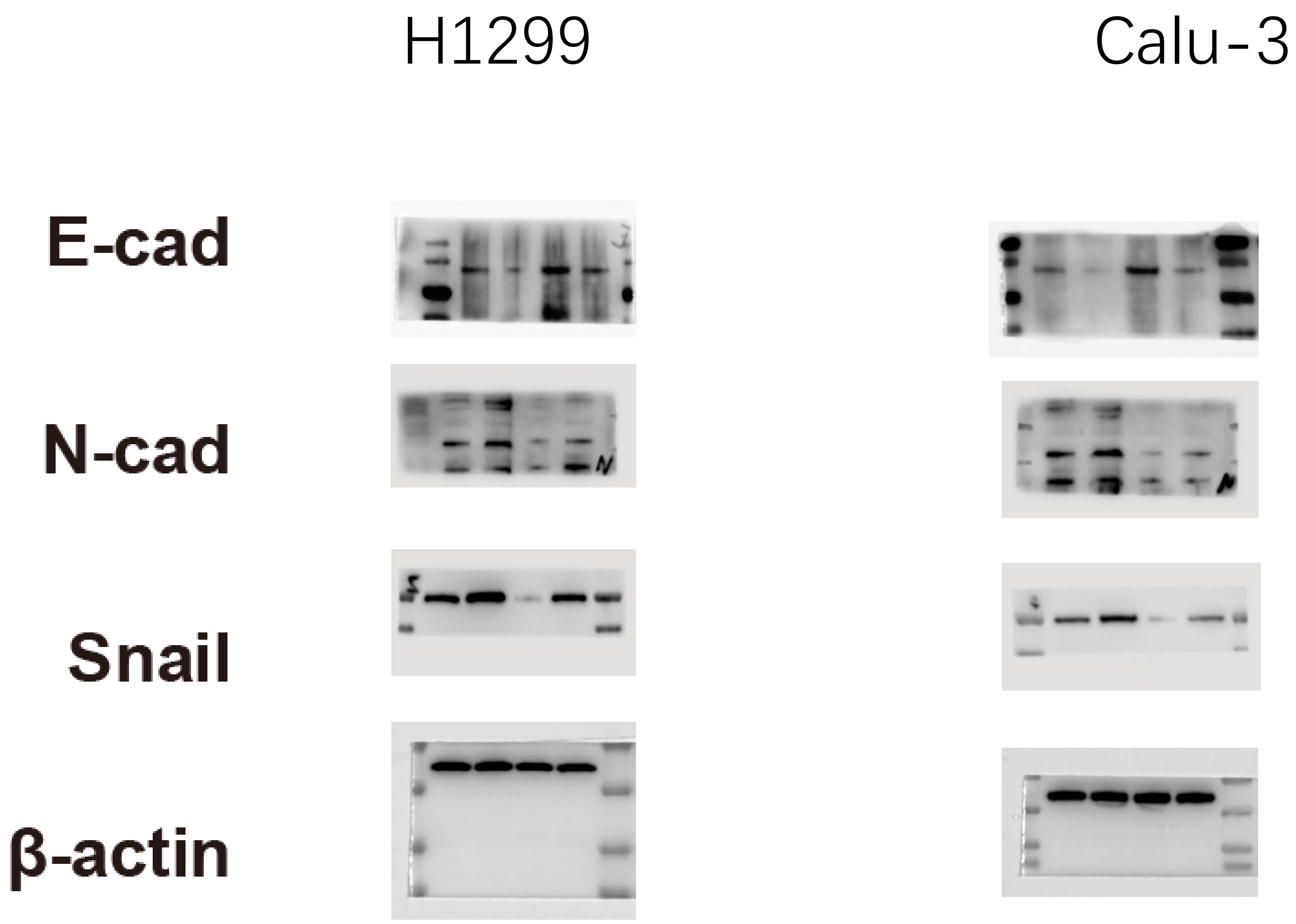

Figure 5C

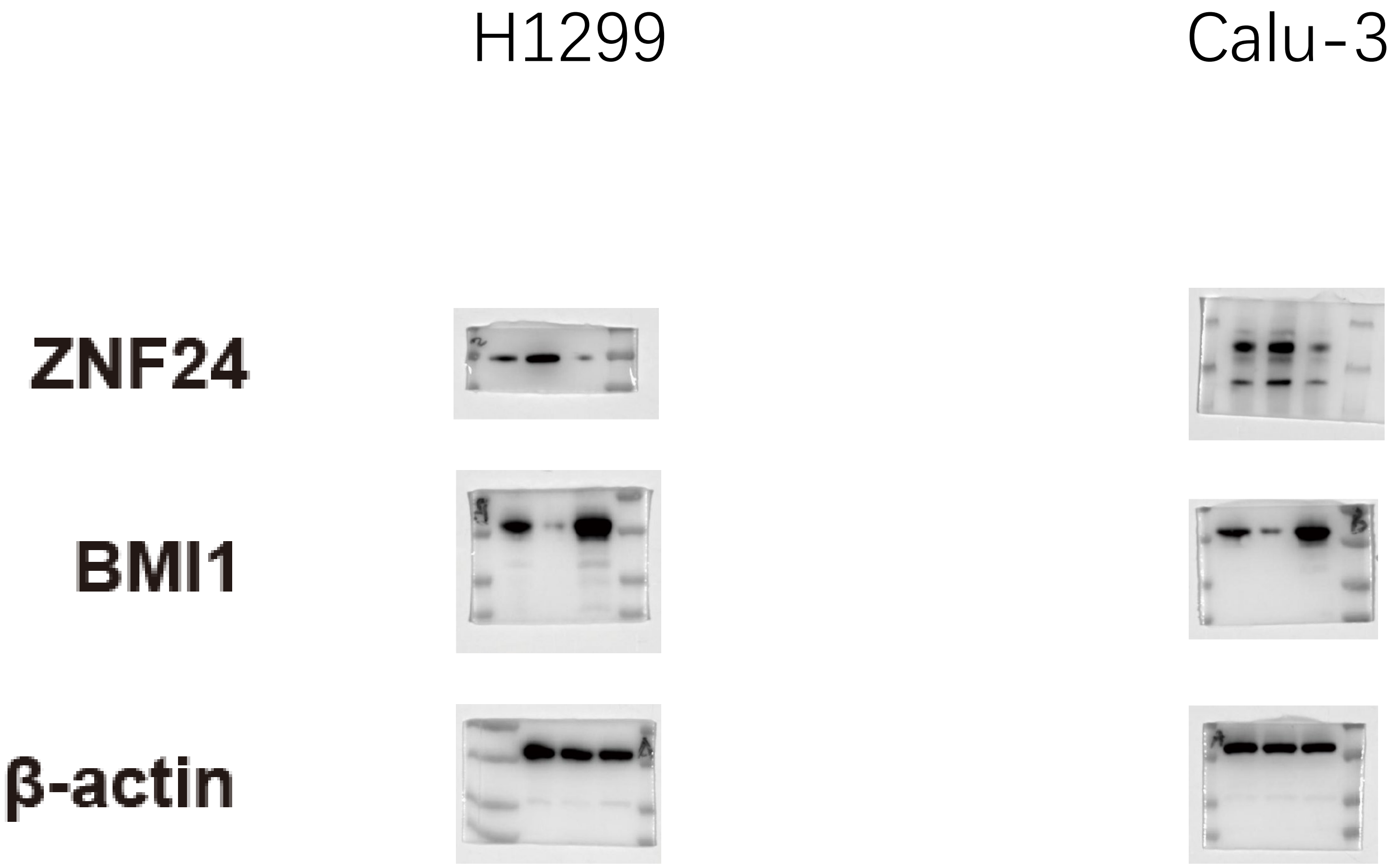

Figure 5E

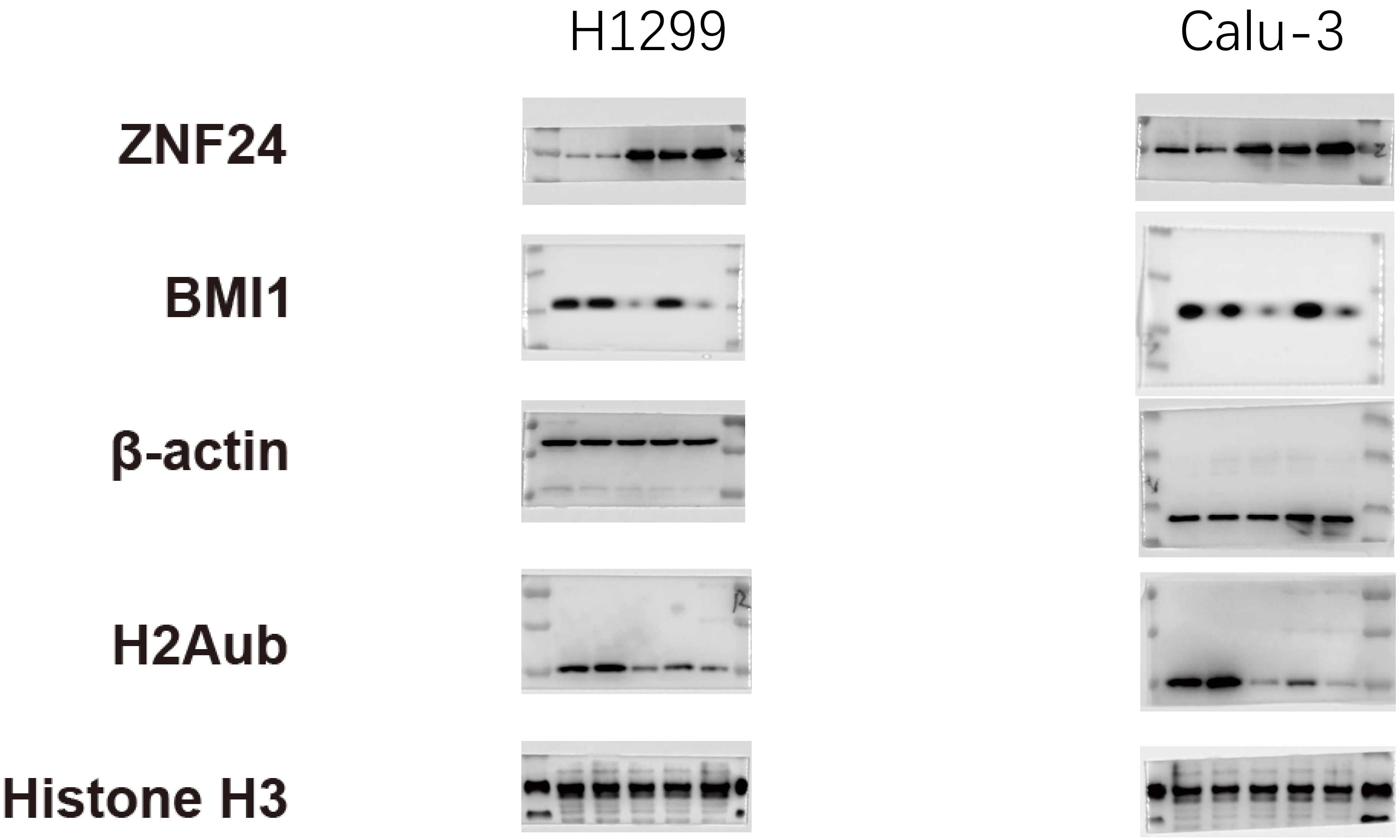

Figure 5I

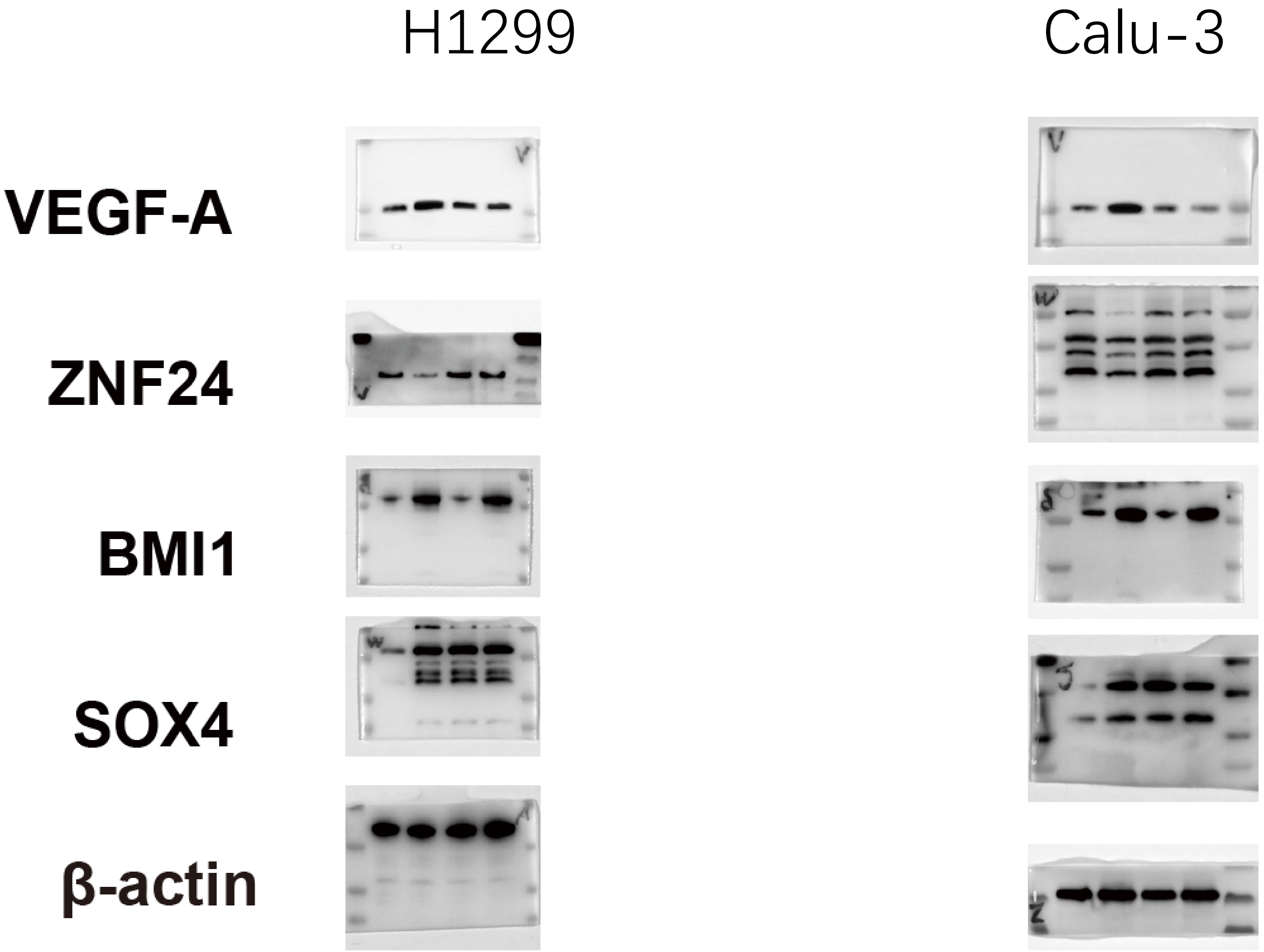

Supplement: Supplementary file 2 — Western Blot Original Image [file 41419_2024_7075_MOESM2_ESM.pdf]
